# Follicle stimulating hormone controls granulosa cell glutamine synthesis to regulate ovulation

**DOI:** 10.1093/procel/pwad065

**Published:** 2024-01-03

**Authors:** Kai-Hui Zhang, Fei-Fei Zhang, Zhi-Ling Zhang, Ke-Fei Fang, Wen-Xing Sun, Na Kong, Min Wu, Hai-Ou Liu, Yan Liu, Zhi Li, Qing-Qing Cai, Yang Wang, Quan-Wei Wei, Peng-Cheng Lin, Yan Lin, Wei Xu, Cong-Jian Xu, Yi-Yuan Yuan, Shi-Min Zhao

**Affiliations:** The Obstetrics & Gynecology Hospital of Fudan University, State Key Laboratory of Genetic Engineering, Fudan University, Shanghai 200090, China; Shanghai Key Laboratory of Metabolic Remodeling, and Children’s Hospital of Fudan University, Shanghai 200032, China; Pediatric Research Institute, Children’s Hospital Affiliated to Shandong University (Jinan Children’s Hospital), Jinan 250022, China; The Obstetrics & Gynecology Hospital of Fudan University, State Key Laboratory of Genetic Engineering, Fudan University, Shanghai 200090, China; The Obstetrics & Gynecology Hospital of Fudan University, State Key Laboratory of Genetic Engineering, Fudan University, Shanghai 200090, China; Shanghai Key Laboratory of Metabolic Remodeling, and Children’s Hospital of Fudan University, Shanghai 200032, China; School of Life Sciences and Institutes of Biomedical Sciences, Fudan University, Shanghai 200438, China; The Obstetrics & Gynecology Hospital of Fudan University, State Key Laboratory of Genetic Engineering, Fudan University, Shanghai 200090, China; Department of Nutrition and Food Hygiene, School of Public Health, Nantong University, Nantong 226019, China; Reproductive Medicine Center, The Affiliated Drum Tower Hospital of Nanjing University Medical School, Nanjing 210008, China; Reproductive Medicine Center, The Affiliated Drum Tower Hospital of Nanjing University Medical School, Nanjing 210008, China; The Obstetrics & Gynecology Hospital of Fudan University, State Key Laboratory of Genetic Engineering, Fudan University, Shanghai 200090, China; The Obstetrics & Gynecology Hospital of Fudan University, State Key Laboratory of Genetic Engineering, Fudan University, Shanghai 200090, China; The Obstetrics & Gynecology Hospital of Fudan University, State Key Laboratory of Genetic Engineering, Fudan University, Shanghai 200090, China; The Obstetrics & Gynecology Hospital of Fudan University, State Key Laboratory of Genetic Engineering, Fudan University, Shanghai 200090, China; The Obstetrics & Gynecology Hospital of Fudan University, State Key Laboratory of Genetic Engineering, Fudan University, Shanghai 200090, China; Department of Animal Science and Technology, Nanjing Agricultural University, Nanjing 210014, China; Key Laboratory for Tibet Plateau Phytochemistry of Qinghai Province, College of Pharmacy, Qinghai University for Nationalities, Xining 810007, China; The Obstetrics & Gynecology Hospital of Fudan University, State Key Laboratory of Genetic Engineering, Fudan University, Shanghai 200090, China; Shanghai Key Laboratory of Metabolic Remodeling, and Children’s Hospital of Fudan University, Shanghai 200032, China; The Obstetrics & Gynecology Hospital of Fudan University, State Key Laboratory of Genetic Engineering, Fudan University, Shanghai 200090, China; Shanghai Key Laboratory of Metabolic Remodeling, and Children’s Hospital of Fudan University, Shanghai 200032, China; Shanghai Fifth People’s Hospital of Fudan University, Fudan University, Shanghai 200240, China; The Obstetrics & Gynecology Hospital of Fudan University, State Key Laboratory of Genetic Engineering, Fudan University, Shanghai 200090, China; The Obstetrics & Gynecology Hospital of Fudan University, State Key Laboratory of Genetic Engineering, Fudan University, Shanghai 200090, China; Shanghai Key Laboratory of Metabolic Remodeling, and Children’s Hospital of Fudan University, Shanghai 200032, China; The Obstetrics & Gynecology Hospital of Fudan University, State Key Laboratory of Genetic Engineering, Fudan University, Shanghai 200090, China; Shanghai Key Laboratory of Metabolic Remodeling, and Children’s Hospital of Fudan University, Shanghai 200032, China; School of Life Sciences and Institutes of Biomedical Sciences, Fudan University, Shanghai 200438, China; Key Laboratory for Tibet Plateau Phytochemistry of Qinghai Province, College of Pharmacy, Qinghai University for Nationalities, Xining 810007, China

**Keywords:** ovulation, glutamine, granulosa cells, PCOS, FSH

## Abstract

Polycystic ovary syndrome (PCOS) is the leading cause of anovulatory infertility. Inadequate understanding of the ovulation drivers hinders PCOS intervention. Herein, we report that follicle stimulating hormone (FSH) controls follicular fluid (FF) glutamine levels to determine ovulation. Murine ovulation starts from FF-exposing granulosa cell (GC) apoptosis. FF glutamine, which decreases in pre-ovulation porcine FF, elevates in PCOS patients FF. High-glutamine chow to elevate FF glutamine inhibits mouse GC apoptosis and induces hormonal, metabolic, and morphologic PCOS traits. Mechanistically, follicle-development-driving FSH promotes GC glutamine synthesis to elevate FF glutamine, which maintain follicle wall integrity by inhibiting GC apoptosis through inactivating ASK1-JNK apoptotic pathway. FSH and glutamine inhibit the rapture of cultured murine follicles. Glutamine removal or ASK1-JNK pathway activation with metformin or AT-101 reversed PCOS traits in PCOS models that are induced with either glutamine or *EsR1*-KO. These suggest that glutamine, FSH, and ASK1-JNK pathway are targetable to alleviate PCOS.

## Introduction

Polycystic ovary syndrome (PCOS) is a leading cause of anovulatory infertility (Azziz et al., 2016). However, owing to the inability to trace the whole process, and a lack of knowledge regarding the factors that signal both oocyte maturation and ovulation, the etiology and pathophysiology of PCOS remain elusive after decades of research. Although genetic variations in nearly 20 genes have been linked to PCOS, a driver gene is yet to be established (Franks and McCarthy, 2004; Mykhalchenko et al., 2017). Endocrine and metabolic abnormalities, including hyperandrogenism and associated traits such as increased serum testosterone and androstenedione levels, high estrogen (Robinson et al., 1992), altered gonadotropin secretion (McCartney et al., 2002), obesity (Legro, 2012), and symptoms of type 2 diabetes (Pelusi et al., 2004), are common in PCOS patients, suggesting that PCOS is an endocrine and/or metabolic disease. However, attempts to induce an animal PCOS model using known hormones or metabolites have had limited success, suggesting that follicle development- and ovulation-driving molecules either fluctuate during follicle development or are distinct from known hormones or metabolites.

Folliculogenesis is promoted by follicle-stimulating hormone (FSH) and luteinizing hormone (LH)—two hormones secreted by the pituitary gland during the menstrual cycle. FSH and LH levels decline during and after ovulation, respectively, and are considered regulators of ovulation. However, it is unclear whether both or one of them plays key ovulation-regulatory roles. A follicle is composed of a multiple-layered wall with outer layers of theca cells (TC) that produce androgen and a much thicker multilayer of granulosa cells (GC). Ovarian steroidogenesis occurs through LH receptors on the theca to produce androgen and through FSH receptors on granulosa cells (Raju et al., 2013) to convert androgen to estrogen, a hormone that is required for oocyte development and maturation (Knobil and Neill, 1998). Ovulation requires a breakdown of the follicle wall, which can be achieved through either outside-in or inside-out manner. Outer TC may be less likely to initiate follicle rupture and ovulation because they are separated from oocytes by multiple layers of GC and may not receive oocyte maturation signals, a prerequisite for ovulation. In contrast, GC, which can communicate with oocytes through follicular fluid (FF), may be good candidates for the initiation of follicle rupture and ovulation because they can receive oocyte maturation signals in FF. Therefore, the apoptotic rate of GC is high in the dominant follicle selection and pre-ovulatory maturation stages of folliculogenesis (Regan et al., 2018), and GC from patients with PCOS exhibit a lower apoptotic rate (Das et al., 2008).

FF contains steroid hormones, proteins, metabolites, and antioxidants (Hildpetito et al., 1991; Selvam et al., 2019) and thus has physiological significance as a source of nutrients for both oocytes and GC. Moreover, FF metabolites may also transmit oocyte maturation and other signals to GC because they are signaling molecules (Chantranupong et al., 2015; Efeyan et al., 2015). To explore whether any molecules in FF may signal ovulation, we screened FF metabolite and found elevated glutamine in the FF of patients with PCOS. We further elucidated how the FSH-regulated FF glutamine controls ovulation by controlling ASK1-JNK-mediated GC apoptosis and proved activating ASK1-JNK pathway can be an intervening strategy for PCOS.

## Results

### GC apoptosis is associated with human PCOS and murine ovulation

**Figure 1.**
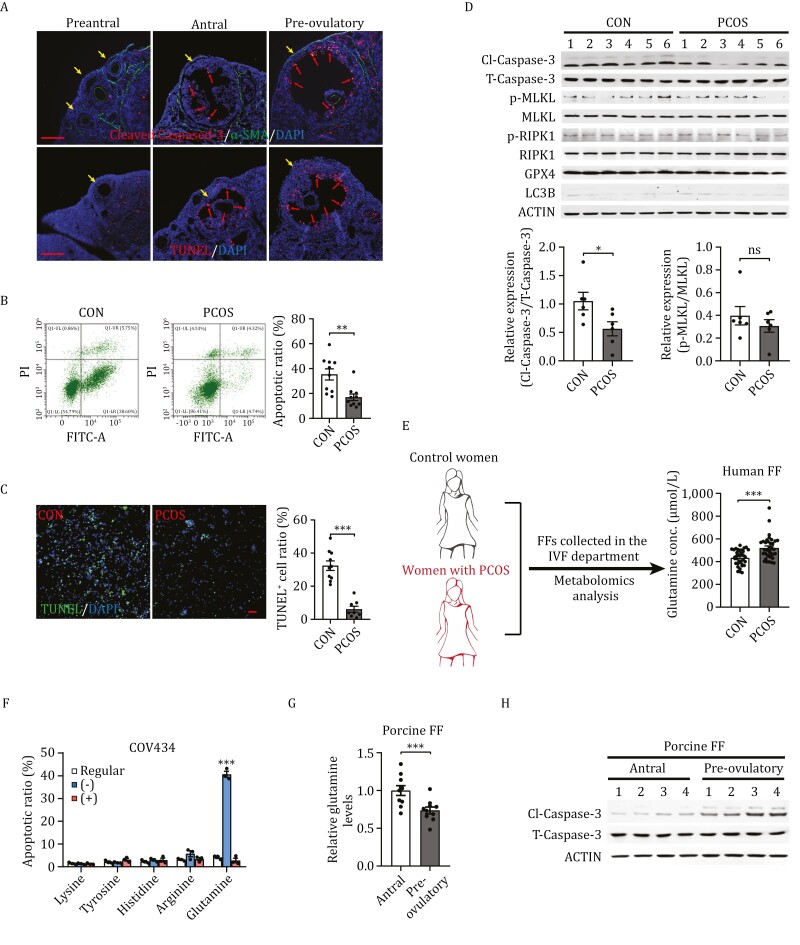
Granulosa cell apoptosis and FF glutamine levels are associated with ovulation outcomes. (A) High FF-facing granulosa cell apoptosis before ovulation. Mouse ovarian sections showing apoptosis of preantral, antral, and pre-ovulatory follicles (yellow arrows) using immunofluorescence (cleaved Caspase-3, upper, red arrows) and TUNEL staining (lower, red arrows) in mouse follicles. α-SMA staining (upper panel) and DAPI staining of theca cells and nuclei, respectively. Scale bars: 100 µm. (B and C) Decreased ratio of apoptotic cells in the FF of patients with PCOS. Apoptosis of GCs in the FF of patients with PCOS and healthy control subjects detected by using flow cytometry (B) and TUNEL staining (C) and then quantified (right). Scale bars: 100 µm, *n* = 10. (D) Apoptosis accounts for the death of floating granulosa cells in the FF. Floating FF cells from patients with PCOS (*n* = 6) and healthy control subjects (*n* = 6) were collected and assayed for markers of apoptosis (Cl-Caspase-3), necrosis (p-RIPK1 and pMLKL), ferroptosis (GPX4), and autophagy (LC3B). The bar graph showed the relative expression of Cl-Caspase-3/T-Caspase-3 (bottom left), and p-MLKL/MLKL (bottom right). (E) Higher glutamine levels were observed in the FF of patients with PCOS than in that of controls. Glutamine levels in the FF of patients with PCOS (*n* = 35) and healthy control subjects (*n* = 37) were quantified using LC–MS/MS. (F) Glutamine starvation increased apoptosis of COV434 cells. Human granulosa COV434 cells were cultured in 1640 medium (regular), 1640 medium with indicated amino acids depleted (–) or 1640 medium with supplemental of indicated extra amino acids (2 mmol/L) for 24 h and cell apoptosis was analyzed using flow cytometry (*n* = 3). (G and H) Decreased porcine FF glutamine levels and GC apoptosis in pre-ovulatory follicles. Glutamine levels in the FF of antral (*n* = 10) and pre-ovulatory (*n* = 10) porcine follicles detected using LC–MS/MS analysis (G). Four samples were randomly selected from each group and analyzed for cleaved Caspase-3 (Cl-Caspase-3) levels using Western blot analysis (H).

To identify ovulation regulators, we compared follicular cell apoptosis in the follicles of C57BL/6 mice at various developmental stages. The apoptosis of GC, but not TC in follicles, increased from the preantral to antral to pre-ovulatory stages, and apoptosis signals were markedly accumulated in FF-exposed GC (Fig. 1A), suggesting that ovulation regulators are present in FF. In human FF samples, flow cytometry (Fig. 1B) and TUNEL assays (Fig. 1C) revealed lower percentages and numbers of floating apoptotic cells in patients with PCOS than those in normal FF, respectively. These results, together with the fact that only the apoptotic marker cleaved Caspase-3 (Cl-Caspase-3), but not the levels of necrosis (p-RIPK1 and pMLKL), ferroptosis (GPX4), and autophagy (LC3B) markers in the FF floating cells of PCOS patients were lower than those in control subjects (Figs. 1D, S1A and S1B), support the hypothesis that apoptosis of FF exposed-GC drives ovulation.

### High glutamine levels in FF of patients with PCOS

To identify the possible ovulation signals in FF, an untargeted metabolomic survey was conducted on the metabolites in the FF of 35 patients with PCOS and 37 age-matched individuals as control (Table S1). Among the metabolites that differed in levels between PCOS and normal FF, several amino acids, including higher glutamine and lower lysine, arginine, histidine, and tyrosine, were found in the FF of patients with PCOS (Figs. 1E, S1C and Table S2). Levels of testosterone (T) (Fig. S1D) were elevated in the FF from PCOS patients, however, levels of estradiol (E2) (Fig. S1E) were comparable between the FF of control and PCOS groups. These results suggest that amino acid dysregulation may regulate ovulation.

Glutamine starvation increased, and glutamine supplementation decreased the apoptosis of cultured human granulosa COV434 cells, as previously reported (Zhang et al., 2000). Altering other amino acid levels had negligible effects on COV434 cell apoptosis (Fig. 1F), suggesting that glutamine possibly regulates ovulation by mediating GC apoptosis. This notion was supported by the fact that pre-ovulatory porcine FF contained lower glutamine (Fig. 1G) but higher Cl-Caspase-3 (Fig. 1H) levels.

### FSH promotes GC glutamine synthesis and FF glutamine levels

**Figure 2.**
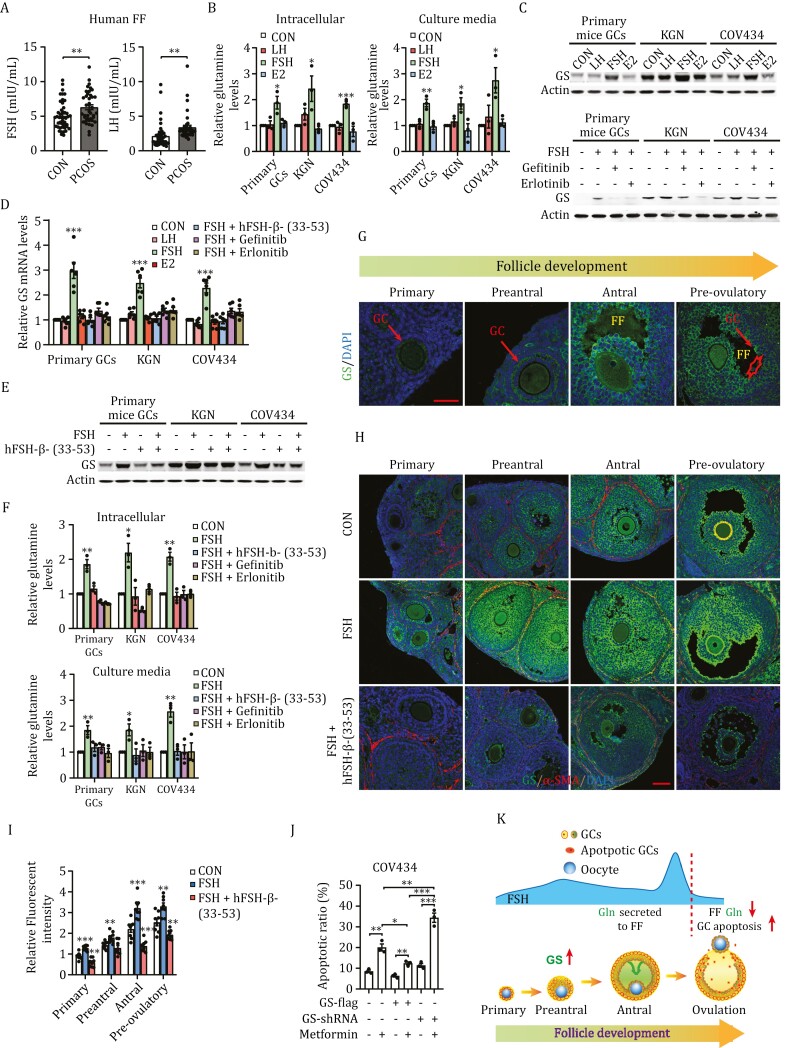
FSH promotes GS expression in GCs and elevates GCs glutamine synthesis and secretion. (A) Higher FSH levels in human PCOS FF. FSH levels in the FF of patients with PCOS (*n* = 40) and healthy control subjects (*n* = 46) were quantified using ELISA. (B) FSH elevates GC glutamine synthesis and secretion. Primary mouse GCs, KGN, and COV434 cells were cultured with LH, FSH, or E2 for 24 h, and the relative glutamine levels in cells and culture medium were measured (described in the Methods section, *n* = 3). (C and D) FSH promotes GS synthesis by activating EGFR-mediated transactivation. Primary mouse GCs, KGN, and COV434 cells were cultured with LH, FSH, or E2 (upper panel) or stimulated with FSH and treated with EGFR inhibitors gefitinib and erlotinib as indicated (lower panel). GS protein levels were detected using Western blot (C) and mRNA levels were quantified using RT-qPCR (D). (E and F) FSH-stimulated glutamine synthesis and secretion were blocked by hFSH-β-(33-53) and EGFR inhibitors. Primary mouse GCs, KGN, and COV434 cells were stimulated with FSH, FSH antagonist, hFSH-β-(33-53), or EGFR inhibitors gefitinib and erlotinib for 24 h. GS protein levels were detected using Western blot (E), and glutamine levels in the intracellular and culture media were quantified (*n* = 3) (F). (G) GS expression in GCs. Immunofluorescence images of mouse follicles at different stages revealed GS expression (green) in GCs that emerged in the preantral follicles and were concentrated in the FF-exposed GCs. Scale bar: 50 μm; (H and I) FSH stimulates GS expression in mice GCs. Eight-week-old female mice were injected with saline (CON), FSH (FSH), or FSH plus hFSH-β-(33-53) (FSH + hFSH-β-(33-53)). Immunofluorescence images of mouse follicles at different stages indicated that FSH upregulated GS expression (green) in GCs, whereas hFSH-β-(33-53) decreased GS expression. α-SMA staining (red) and DAPI staining (blue) were performed on theca cells and nuclei, respectively (H). Scale bar: 50 µm. The relative fluorescence intensity in the GCs was analyzed using Image J (I) (*n* = 8 in each group). (J) GS regulates metformin-induced apoptosis. COV434 cells were transfected with GS-flag or GS shRNA and treated with metformin for 24 h, as indicated. GS-overexpressing and GS-knockdown COV434 cells were resistant and sensitive to metformin-induced apoptosis, respectively (*n* = 3). (K) Schematic diagram of the FSH controls the GS and FF glutamine levels in GC during follicle development and ovulation. FSH stimulates GS expression in GC at the preantral stage and maintains its expression throughout follicular development. FF-exposed GC, which have the highest GS level, actively synthesize and secrete glutamine to the FF to nourish the oocyte and sustain follicle wall. Before ovulation, the rapid decrease in FSH levels results in low FF glutamine levels and apoptosis of GCs, starting from FF-exposed GCs. This promotes the breakup of the follicle wall, leading to successful ovulation.

While tracing the origin of FF glutamine, we found that both FSH and LH levels were higher in the FF of patients with PCOS (Fig. 2A). Moreover, FSH, but not LH, increased glutamine levels intracellularly and in the culture media of primary mice GCs, KGN cells, and COV434 cells (Fig. 2B). These results suggest that FSH drives glutamine synthesis and secretion. FSH upregulated glutamine synthetase (GS), an enzyme that synthesizes glutamine from glutamate, protein levels (Fig. 2C), via upregulating *GS* transcription (Fig. 2D) in these cells. These, together with the epidermal growth factor receptor (EGFR) inhibitors erlotinib and gefitinib, which bind to the tyrosine kinase domain and stop the activity of EGFR (Yang et al., 2017), inhibit the ability of FSH to upregulate *GS* transcription (Fig. 2D) and protein (Fig. 2C) levels. Mimicking FSH decrease at the pre-ovulation stage with FSH receptor binding inhibitor hFSH-β-(33-53) (TFA) (Santa-Coloma et al., 1992) decreased GS levels (Fig. 2E) and glutamine levels in both cells and culture media (Fig. 2F) of FSH-stimulated COV434 cells, confirming that FSH promotes glutamine synthesis by promoting GS expression.

Immunofluorescence staining of mouse ovaries revealed that in primary follicles, GS was expressed mainly in oocytes and cumulus oophorus cells; GS expression in GCs, which have the same origin as cumulus oophorus cells, emerged in secondary follicles and, remarkably, GS expression was concentrated in the FF-exposed GC, and this trend was maintained throughout follicle development until the follicle rupture, in which GS expression diminished in the apoptotic GCs (Fig. 2G). Moreover, subcutaneous FSH injection in female mice promoted GS expression in GCs (Figs. 2H and 2I), consistent with that the GS expression was dependent on FSH stimulation (Fig. 2F). Furthermore, *GS*-overexpressing and *GS*-knockdown rendered COV434 cells resistant and sensitive to metformin-induced apoptosis, respectively (Fig. 2J). These results verify the hypothesis that FSH, which enhances GS expression by activating EGFR-mediated transactivation, controls GC GS and FF glutamine levels during follicle development and ovulation (Fig. 2K).

### Glutamine inhibits GC apoptosis via ASK1-JNK apoptotic pathway

**Figure 3.**
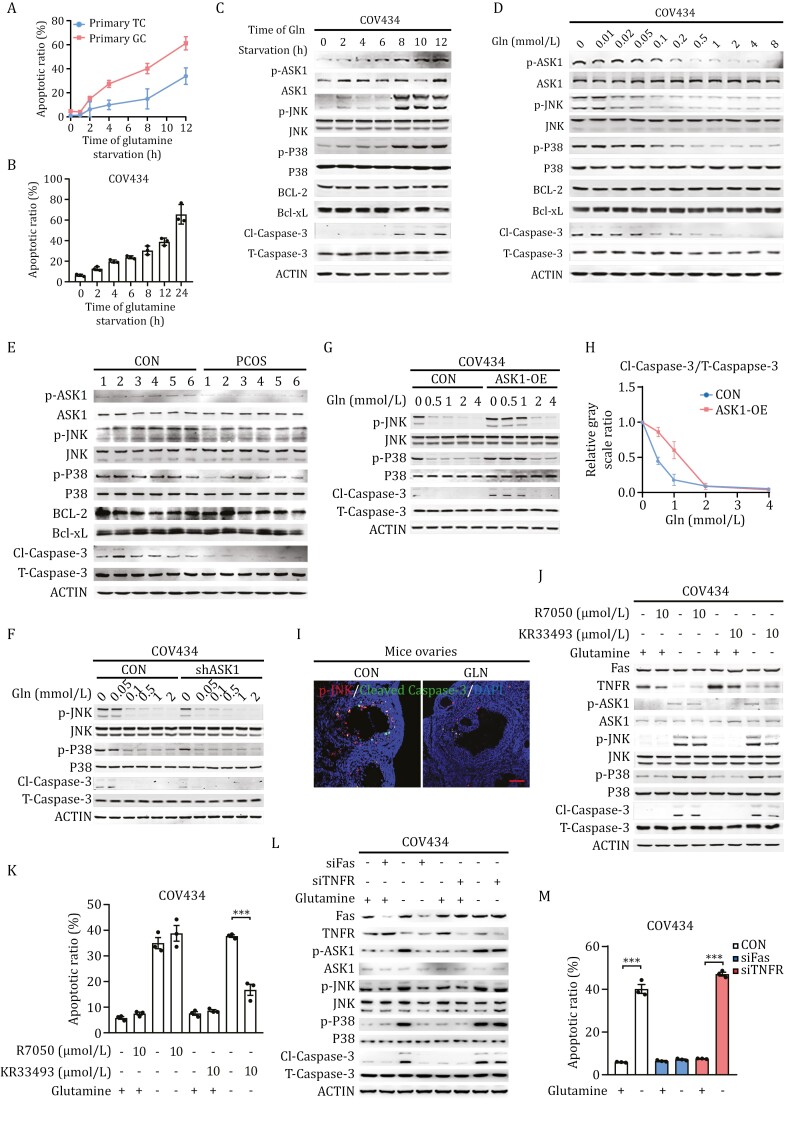
Glutamine inhibits GC apoptosis via ASK1 apoptotic signaling pathway. (A) Glutamine starvation specifically induces apoptosis of mice granulosa cells. Primary mouse TCs and GCs were cultured in McCoy’s 5A medium and then changed to a glutamine-free 1640 medium. Cell apoptotic ratios were measured at the indicated time points using flow cytometry (*n* = 3). (B) Glutamine starvation induces apoptosis in granulosa cells. Flow cytometry analysis (*n* = 3) of the apoptotic ratio of granulosa cells at 0, 2, 4, 6, 8, 12, and 24 h after glutamine deprivation in COV434 cells. (C–H) Glutamine deprivation induces GC apoptosis by activating ASK1 signaling. ASK1 and its downstream molecular pathways, JNK and P38, were activated during glutamine deprivation in a time-dependent manner (C), and inhibited by glutamine supplementation (D), as well as in the FF floating cells of PCOS patients (E). ASK1 knockdown reversed apoptotic signaling induced by glutamine deficiency (F), whereas overexpression of ASK1 promoted apoptotic signaling in COV434 cells (G and H). (I) JNK activation in mice follicles. Immunofluorescence staining of p-JNK (red) and cleaved Caspase-3 (green) with DAPI (blue) in mouse ovaries. Scale bar: 50 μm. (J–M) Fas-signaling rather than TNFR signaling mediates granulosa cell apoptosis. Inhibition of Fas signaling by KR33493, but not TNF-α signaling by R7050, attenuates JNK and P38 phosphorylation (J) and apoptosis (K) induced by glutamine deprivation. Consistent with this, Fas-knockdown, but not TNFR-knockdown, COV434 cells inhibited JNK and P38 phosphorylation (L) and resistance to apoptosis (M) induced by glutamine deprivation (*n* = 3).

To investigate how GC apoptosis is regulated by FF glutamine, we starved the primary cultured ovarian GCs for glutamine to mimic glutamine reduction in FF during ovulation, employing theca cells, another type of cell in the follicle wall, as a control. GCs were more sensitive to glutamine starvation than theca cells (Fig. 3A), further supporting that glutamine deprivation specifically induces GC apoptosis during ovulation. Consistent with this, glutamine starvation-induced apoptosis in COV434 (Figs. 3B and S2A) and KGN (Fig. S2B) cells, two widely used human granulosa cell lines (Zhang et al., 2000; Nishi et al., 2001), in a time-dependent manner. Moreover, Jun N-terminal protein kinase (JNK) and P38, downstream targets of ASK1 that are inactivated by glutamine (Ko et al., 2001), were activated by glutamine starvation in a time-dependent manner, whereas BCL-2 and Bcl-xL, two key molecules of the intrinsic apoptotic pathway, remained unaffected by glutamine deprivation (Figs. 3C and S2C). These results suggest that the death receptor- and ASK1-mediated extrinsic apoptotic pathway, rather than the mitochondrial-mediated intrinsic apoptotic pathway (Sasaki et al., 2009), governs the glutamine-regulated apoptosis of GCs. This hypothesis is further supported by the following observations: (i) glutamine supplementation inhibited the phosphorylation of ASK1, JNK, and P38, but had negligible effects on BCL-2 and Bcl-xL in both COV434 and KGN cells (Figs. 3D and S2D); (ii) in the FF floating cells from PCOS patients, which were immersed in higher glutamine environment, ASK1-JNK apoptotic signaling, but not BCL-2 and Bcl-xL, was significant attenuated (Fig. 3E); (iii) ASK1 knockdown using short hairpin RNA in COV434 cells (Fig. S2E) did not affect the phosphorylation levels of JNK and P38 (Fig. 3F); (iv) overexpression of ASK1 in COV434 cells (Fig. S2F), enhanced the ability of glutamine to regulate JNK and P38 phosphorylation (Figs. 3G and 3H). Furthermore, phosphorylation of JNK, which is co-localized with cleaved Caspase-3, was noticed in the inner layer of GCs exposed to FF (Fig. 3I).

We previously found that glutamine suppresses ASK1-mediated apoptosis through glutaminyl-tRNA synthetase (QARS)-catalyzed ASK1 K688 glutaminylation (He et al., 2018). To investigate whether FF glutamine depletion induces GC apoptosis via the same mechanism, we tested the effects of glutamine on QARS knockdown (Fig. S3A) and QARS overexpressing (Fig. S3B) COV434 cells. QARS knockdown reduced the ability of glutamine to suppress ASK1 signaling in a dose-dependent manner (Fig. S3C), and potentiated glutamine deprivation to activate ASK1 signaling (Fig. S3D), whereas QARS overexpression increased the ability of glutamine to suppress ASK1 signaling (Fig. S3E). These results are consistent with the hypothesis that FF glutamine depletion initiates GC apoptosis by activating ASK1 apoptotic signaling through QARS-mediated glutamine signaling.

To further address the upstream receptor that transduces death signaling of glutamine starvation to the extrinsic ASK1-mediated apoptotic pathway, we knocked down the two main death receptors using siRNA or blocked their functions with specific inhibitors. Unlike the TNFR antagonist R7050, KR33493, an inhibitor of ASK1-mediated apoptosis through Fas-signaling inhibition (Jeong et al., 2016), inhibited JNK and P38 phosphorylation (Figs. 3J and S3F) and apoptosis (Figs. 3K and S3G) induced by glutamine deprivation in COV434 and KGN cells. Consistent with this, knockdown of Fas, but not TNFR, inhibited the ASK1-mediated extrinsic apoptotic pathway and apoptosis (Figs. 3L and 3M). Together, these results suggest that GC apoptosis is specifically mediated by ASK1-JNK signaling.

### Glutamine and FSH regulates ovulation *in vitro*

**Figure 4.**
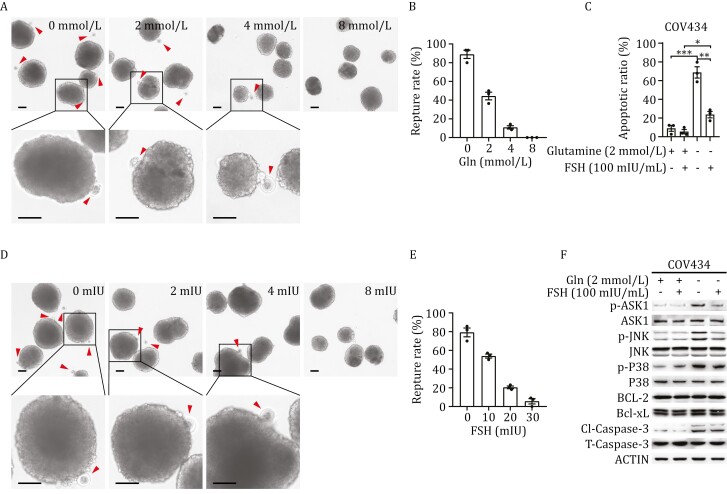
Glutamine and FSH regulates ovulation ***in vitro***. (A and B) Glutamine inhibits mice follicular rupture *in vitro*. Mice follicles were cultured to mature *in vitro*, and were induced to ovulation, indicated by follicular rapture (enlarged below), by hCG with the presence of different levels of glutamine. Images were taken after 16 h after hCG induction. Red arrows indicate the ovulated eggs (A), and the follicular rupture ratio for each group was statistically calculated (*n* = 3) (B). Bars: 100 μm. (C) FSH sustains cell survival under glutamine starvation. COV434 cells were treated with or without 100 mIU FSH under glutamine-rich or glutamine-free medium for 8 h, the apoptotic rate of cells was measured with flow cytometry (*n* = 3). (D and E) FSH inhibits mice follicular rupture *in vitro*. Mice follicles were cultured to mature *in vitro*, and were induced to ovulation by hCG with presence of different levels of FSH. Follicular rapture was visualized 16 h after hCG induction (D), and the follicular rupture ratio for each group was statistically calculated (*n* = 3) (E). Bars: 100 µm. (F) FSH inhibits ASK1-JNK apoptotic signaling. COV434 cells were treated with or without 100 mIU FSH under a glutamine-rich or glutamine-free medium for 8 h, and the ASK1-JNK apoptotic signaling was detected by Western blot.

We tested their efficacies of FSH and glutamine on isolated murine follicles. Glutamine dose-dependently inhibited follicular rupture of cultured murine follicles (Figs. 4A and 4B). Moreover, FSH administration decreased the apoptosis of granulosa cells with glutamine starvation (Fig. 4C) and inhibited follicular rupture of cultured murine follicles (Figs. 4D and 4E). Furthermore, FSH treatment, which stimulates glutamine synthesis, weakens the ASK1-JNK apoptotic signaling in cultured granulosa cells (Fig. 4F). These solidify that FSH and it-promoted FF glutamine directly regulate ovulation rather than regulating ovulation through alternative mechanisms such as changing other ovulation-relative hormones/signaling.

### High glutamine induced PCOS traits

**Figure 5.**
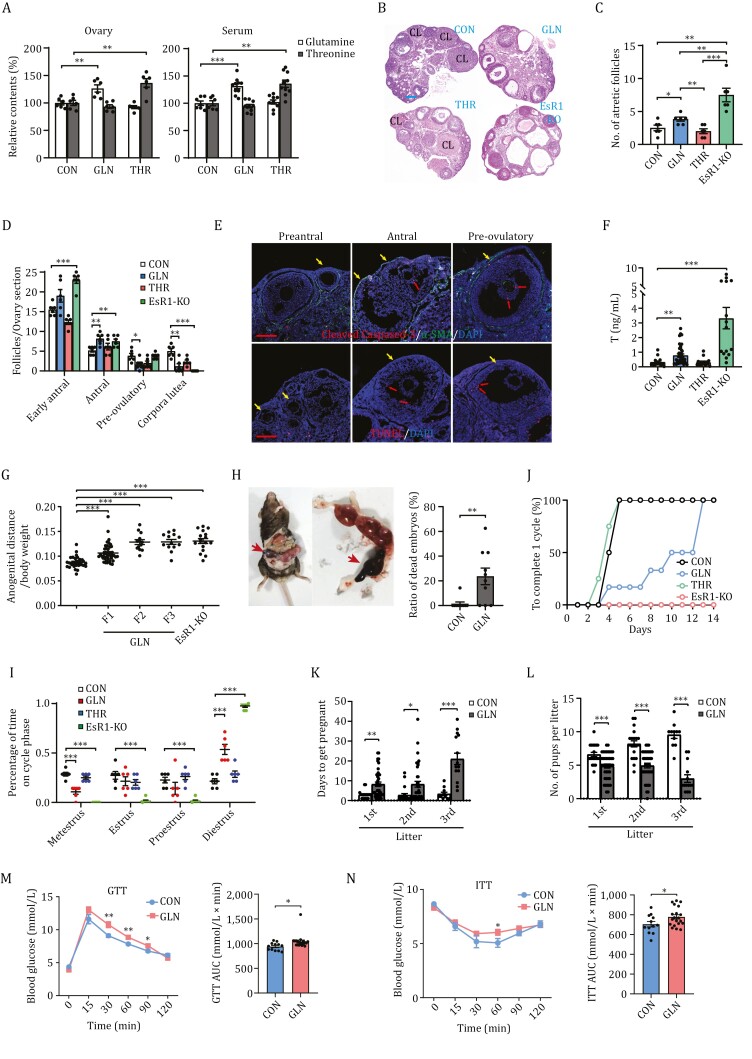
High-glutamine chow induces PCOS traits in mice. (A) High-amino acids chow feed increases mice ovary and serum amino acids levels. Ovaries and serum glutamine and threonine levels in mice that were fed a high-glutamine (GLN) or high-threonine chow (THR) were measured using LC–MS/MS analysis. Ovaries: *n* = 6 per group; serum: *n* = 6–10 per group. (B) High-glutamine chow induces PCOS morphology in female mice. Hematoxylin and eosin staining of ovaries from representative control (CON), high-glutamine chow (GLN), high-threonine chow (THR), and EsR1-KO mice (EsR1-KO). CL, corpus luteum. Scale bar: 100 µm. (C and D) High-glutamine chow induces fewer pre-ovulatory follicles and corpora lutea. Mean atretic follicle numbers in CON, GLN, THR, and EsR1-KO mouse ovaries (C) (*n* = 6 per group), and quantitative analysis of early antral follicles, antral follicles, pre-ovulatory follicles, and corpus luteum in CON, GLN, THR, and EsR1-KO mouse ovaries (D) (*n* = 6 per group). (E) Lower FF-facing granulosa cell apoptosis in high-glutamine fed mice (GLN) ovaries. GLN mice ovarian sections were detected for apoptosis of preantral, antral, and pre-ovulatory follicles (yellow arrows) using immunofluorescence (cleaved Caspase-3, upper, red arrows) and TUNEL staining (lower, red arrows) in high-glutamine chow-fed mouse follicles.α-SMA staining (upper panel) and DAPI staining of theca cells and nuclei, respectively. Scale bars: 100 µm. (F–L) High-glutamine chow induces PCOS traits in mice. Serum testosterone (F), ratio of anogenital distance to body weight in F1, F2, and F3 female mice and in EsR1-KO mice (G) (CON: *n* = 30; GLN: *n* = 13–44; EsR1-KO: *n* = 17), absorbed embryos during pregnancy (H), percentage of time on estrous cycle phases of female mice (I), days to complete one estrous cycle (J), determined for CON, GLN, THR, and EsR1-KO mice as indicated. And average time to get pregnant for the first, second, and third litter (K), and litter pup numbers for the first, second, and third litter (L) determined for CON, and GLN mice as indicated (CON: *n* = 11–21; GLN: *n* = 14–41). (M and N) High-glutamine chow impairs glucose and insulin tolerance in female mice. GTT (CON: *n* = 12; GLN: *n* = 20) (L) and ITT (CON: *n* = 12; GLN: *n* = 20) (M) of the CON and GLN mice, and the areas under the curve were statistical analyzed and presented as a bar graph, respectively.

Next, we investigated whether high FF glutamine levels, but not that of other amino acids such as threonine, are a causal factor of PCOS. Either high-glutamine or high-threonine chows were fed to 5-week-old C57BL/6 mice (designated as GLN mice and THR mice, respectively), which increased ovary and serum glutamine levels by approximately 30%–40% that mimicked the elevated FF glutamine levels found in PCOS, and a similar degree of threonine elevation, respectively, compared to those of normal chow-fed C57BL/6 mice (designated as CON mice) after 3 months of treatment (Fig. 5A).

Physiological markers and PCOS traits were compared among mice fed with different chow (Fig. S4A). Both GLN and THR mice consumed less (Fig. S4B), and exhibited lower body weights (Fig. S4C) than that of CON mice, which is consistent with the findings of a previous study showing that a high-protein diet decreases body weight (Westerterp-Plantenga et al., 2004). The GLN and THR mice also consumed more water (Fig. S4D) and excreted more urine than that observed for CON mice (Fig. S4E), which is likely because high glutamine and threonine diets produce more ammonia that must be secreted in the form of urine.

GLN mouse ovaries phenocopied the ovaries from estrogen receptor 1 knockout (EsR1-KO) mice and dihydrotestosterone (DHT) induced PCOS mice, two known PCOS mice models (Lee et al., 2009; Rodriguez Paris et al., 2020) (Figs. 5B and S5A), to exhibit an increased number of atretic (Fig. 5C), early and antral follicles, and fewer pre-ovulatory follicles and corpora lutea (Fig. 5D) than those of CON mice, whereas THR mouse ovaries were indistinguishable from those of CON mice (Figs. 5B and 5D). Notably, reduced GC apoptosis, and thicker and more condensed GC layers were observed in the follicles of GLN mice (Fig. 5E), which is consistent with the hypothesis that high glutamine-induced insufficient GC apoptosis may promote PCOS traits.

Unlike THR mice, GLN mice recapitulated the phenotypes of androgen-induced PCOS mice (Risal et al., 2019), resulting in higher serum testosterone levels (Fig. 5F) than in CON mice. Moreover, a longer anogenital distance in F1, F2, and F3 female mice (Fig. 5G) and more dead embryos were found in GLN mice (Fig. 5H), which further confirmed high testosterone exposure in GLN mice. Furthermore, only GLN mice simulated EsR1-KO mice and DHT mice to induce irregular estrous cycles (Figs. 5I and S5B), had a longer estrous cycle (Figs. 5J and S5C), took longer to become pregnant (Fig. 5K), and had smaller litter pup numbers (Fig. 5L). Finally, moderate impairment of glucose tolerance and insulin tolerance was observed in GLN mice according to the glucose (GTT) and insulin tolerance test (ITT) assays, respectively (Figs. 5M and 5N), which is consistent with the observed reduction in the systemic insulin sensitivity mediator adiponectin (Fig. S5D) and a relatively normal basal level of insulin (Fig. S5E) in GLN mice. Additionally, GLN mice exhibited a lower subcutaneous fat mass (Fig. S5F) and smaller adipocyte sizes (Fig. S5G), which was consistent with the lower lipid storage in the liver (Fig. S5H), thereby refuting the possibility that glutamine induces PCOS symptoms by increasing the body fat. Collectively, these results indicate that high ovarian glutamine levels may induce PCOS traits.

Notably, although GLN mice exhibited an increase in blood testosterone (Fig. 5F), no statistically significant differences were observed in the levels of E2 (Fig. S5I), ovulation-promoting LH (Fig. S5J), and follicle-stimulating FSH (Fig. S5K), all of which are dysregulated by testosterone, suggesting that glutamine may perform other actions on these hormones, in addition to increasing testosterone levels.

### Alleviating PCOS through glutamine deprivation

**Figure 6.**
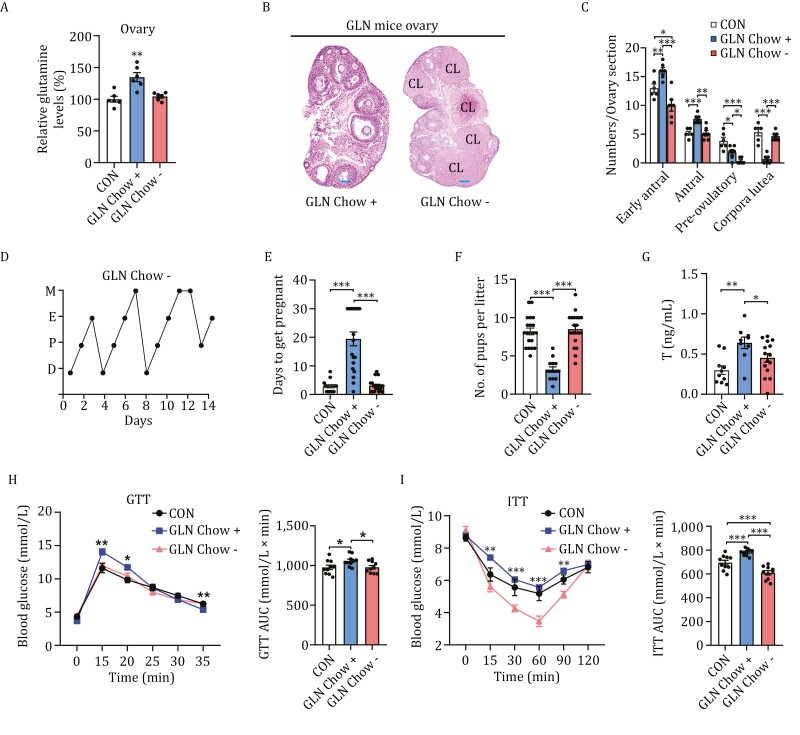
Glutamine removal reverses PCOS traits in GLN mice. (A–C) Glutamine chow removal induces successful ovulation. Relative glutamine levels in mouse ovaries (A, *n* = 6 per group) and hematoxylin and eosin staining for ovaries assayed for normal chow-fed (CON), glutamine chow-fed (GLN chow+) mice, and mice fed glutamine chow for three months followed by normal chow feeding for one month (GLN chow-) (B). Scale bars: 100 µm (*n* = 6 per group; representative images are shown; CL: corpus luteum). Quantitative analysis of early antral follicles, antral follicles, pre-ovulatory follicles, and CL in CON, GLN chow+, and GLN chow- mouse ovaries (C). (D–F) Glutamine chow removal restored the regular estrous cycle and litter pup number. Representative estrous cycles (D), time to get pregnant (E, *n* = 20 per group), and litter pup numbers (F, *n* = 20 per group) of CON, GLN chow+, and GLN chow- mice were measured. (G) Glutamine chow removal decreases serum testosterone levels. Serum testosterone levels in CON (*n* = 10), GLN chow + (*n* = 9), and GLN chow- (*n* = 15) mice. (H and I) Glutamine chow removal alleviates mice’s glucose tolerance and insulin resistance. Glucose tolerance test (H) and insulin resistance test (I) results for CON, GLN chow+, and GLN chow- mice (*n* = 10 per group), and the areas under the curve were statistical analyzed and presented as bar graphs, respectively.

To further confirm that FF glutamine signals oocyte maturation and promotes GC apoptosis and ovulation, we examined the effects of glutamine removal in GLN mice. Glutamine was removed from GLN mice by feeding the mice normal chow for one month, which reduced their ovarian glutamine levels to levels comparable to those of CON mice (Fig. 6A). Glutamine removal was accompanied by fewer antral follicles and more corpus luteum in the ovaries of GLN mice (Figs. 6B and 6C), suggesting that removal of high-glutamine chow promoted ovulation. This finding was further substantiated by the observation that the removal of high-glutamine chow restored the regular estrous cycle of GLN mice (Fig. 6D), shortened the time to get pregnant (Fig. 6E), and increased the litter pup number to levels comparable to those of CON mice (Fig. 6F). Moreover, the removal of glutamine chow decreased the blood testosterone levels of GLN mice (Fig. 6G), but had negligible effects on E2 (Fig. S5L), FSH (Fig. S5M), and LH (Fig. S5N), which was consistent with the finding that glutamine chow did not alter these hormones (Figs. S5I–K) and the hypothesis that glutamine may have additional effects on hormones other than testosterone. Furthermore, the removal of the high-glutamine diet decreased glucose tolerance in GLN mice (Fig. 6H) and increased their sensitivity to insulin (Fig. 6I). Together, these results confirm that dynamic regulation of glutamine levels is associated with ovulation and PCOS traits.

### Targeting Fas-ASK1 apoptotic pathway to alleviate PCOS

**Figure 7.**
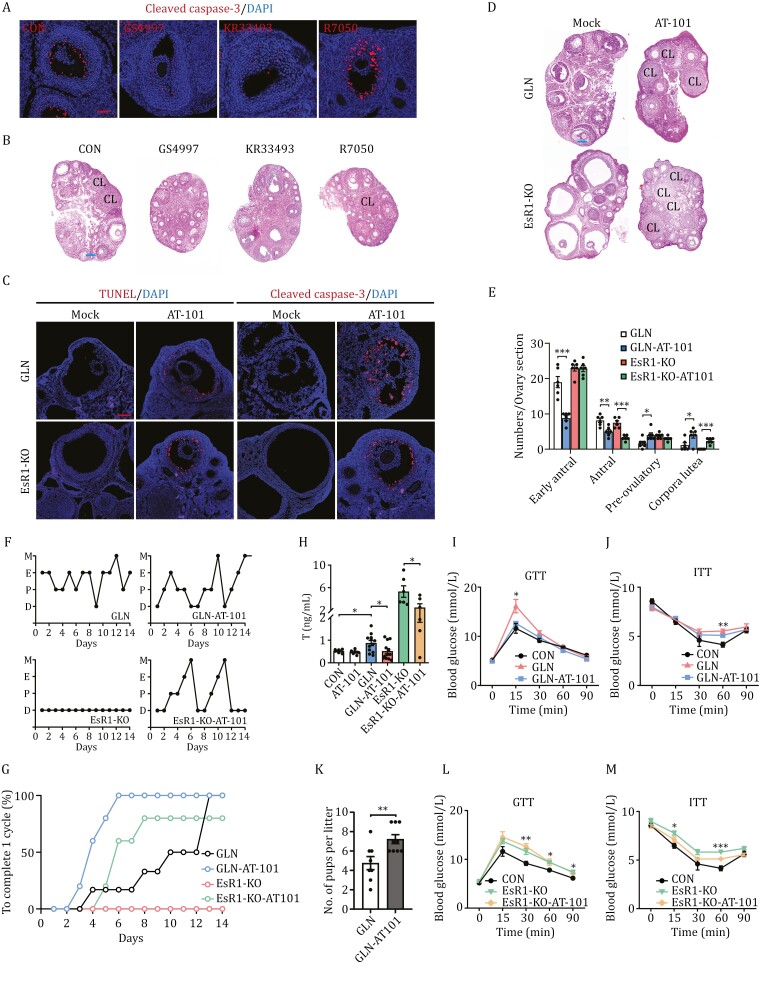
ASK1 signaling activation alleviates PCOS traits in mice PCOS models. (A) Inhibition of Fas-ASK1 death signaling decreases the apoptosis in GCs of mice ovaries. Immunofluorescence staining of cleaved caspase-3 (red) with DAPI (blue) staining of ovaries from mice treated with GS4997, KR33493, and R7050. Scale Bar: 100 µm. (B) Inhibition of Fas-ASK1 death signaling induces polycystic ovary morphology in mice. H&E staining of ovaries from mice treated with saline (CON), GS4997, KR33493, and R7050. CL: corpus luteum. Scale Bar: 100 µm. (C–E) AT-101 increases granulosa cell-specific apoptosis and ovulation in mice. TUNEL staining (C, left), immunofluorescence staining (C, right), and hematoxylin and eosin staining (D) of ovaries from GLN or EsR1-KO mice fed via oral gavage of saline (Mock) or AT-101 (AT-101). Scale bars: 100 µm. (E) Quantitative analysis of early antral follicles, antral follicles, pre-ovulatory follicles, and corpus luteum per ovary section in GLN, GLN-AT-101, EsR1-KO, and EsR1-KO-AT-101 ovaries (*n* = 6 per group). (F and G) AT-101 partially reverses PCOS mice model estrous cycles. Representative estrous cycles of GLN, GLN-AT-101, EsR1-KO, and EsR1-KO-AT-101 females (F) and days taken for GLN, GLN-AT-101, EsR1-KO, and EsR1-KO-AT-101 mice to complete one estrous cycle (G). (H) AT-101 decreases serum testosterone levels in GLN mice and EsR1-KO mice. Serum testosterone levels in CON, AT-101, GLN, GLN-AT-101, EsR1-KO, and EsR1-KO-AT-101 mice (*n* = 6–12 per group). (I and J) AT-101 treatment increases glucose tolerance and insulin sensitivity in GLN mice. GTT (I) and ITT (J) of CON, GLN, and GLN mice injected with AT-101 (CON and GLN: *n* = 6; GLN-AT-101: *n* = 5). (K) AT-101 treatment increases the number of pups in GLN mice. Litter pup numbers in GLN and GLN mice treated with AT-101 (*n* = 8–9). (L and M) AT-101 treatment increases glucose tolerance and insulin sensitivity in EsR1-KO mice. GTT (L) and ITT (M) of CON, EsR1-KO, and EsR1-KO mice injected with AT-101 (CON: *n* = 6; EsR1-KO and EsR1-AT-101: *n* = 5).

To verify that Fas-ASK1-mediated apoptotic pathway plays a critical role in follicular maturation and ovulation, female mice were treated with compounds that inhibit the Fas-ASK1 signaling pathway. Oral gavage of KR33493 and GS4997, which inhibit Fas and ASK1, respectively, induced PCOS-like symptoms in mice, such as polycystic ovary morphology (Figs. 7A and 7B), and elevated levels of serum testosterone (Fig. S6A), but not of estrogen (Fig. S6B).

To further confirm that GC apoptosis drives ovulation, we treated C57BL/6 female mice with AT-101, a compound that activates the ASK1/JNK pathway-mediated apoptosis (Zerp et al., 2009). AT-101 specifically induced GC apoptosis in GLN mouse follicles (Figs. 7C, S6C and S6D), confirming that GC apoptosis is specifically regulated by the ASK1-JNK apoptotic pathway. Moreover, oral gavage of AT-101 stimulated ovulation, as shown by the increased number of corpora lutea (Figs. 7D and S6G). AT-101 treatment consistently reduced the number of early antral and antral follicles, increased the number of pre-ovulatory follicles (Fig. 7E), restored regular estrous cycles in GLN mice (Figs. 7F and 7G), decreased serum testosterone (Fig. 7H) but not estradiol (Fig. S6I), and increased glucose tolerance (Figs. 7I, and S6J) and insulin sensitivity (Figs. 7J and S6K). Furthermore, AT-101 treatment increased the number of pups in GLN mice (Fig. 7K). Together, these results confirmed that AT-101 attenuates the PCOS-induced effects of glutamine.

If insufficient GC apoptosis is the cause of anovulation, AT-101 treatment should also alleviate PCOS traits induced through other mechanisms. We tested this hypothesis by applying AT-101 treatment to an EsR1-KO-induced PCOS mice model (Walters et al., 2012). AT-101 treatment induced GC-specific apoptosis (Figs. 7C, S6E and S6F), increased the number of corpora lutea (Figs. 7Dand S6H), decreased the number of early antral and antral follicles, increased the number of pre-ovulatory follicles (Fig. 7E), restored regular estrous cycles in EsR1-KO mice (Figs. 7F and 7G), decreased serum testosterone levels (Fig. 7H), and increased glucose tolerance (Figs. 7L and S6L) and insulin sensitivity (Figs. 7M and S6M). Notably, AT-101 decreased the number of hemorrhagic cysts in the ovaries of EsR1-KO mice (Fig. S6N). Given that EsR1-KO mice had the same normal serum glutamine levels as control mice (Fig. S6O), these results confirmed that the induction of GC apoptosis alleviated PCOS traits.

## Discussion

In the current study, by metabolic, cell biologic, *in vitro* and *in vivo* approaches, we found that FSH, the hormone that promotes GC proliferation and initiates follicle development for oocyte maturation, plays an additional role in regulating GC glutamine synthesis for ovulation. Glutamine plays a plethora roles in oocyte/follicular development/maturation. First, stemness oocyte maturation inside a follicle prefers glutamine as a nutrient source (Vardhana et al., 2019). FSH-facilitated glutamine synthesis thus supplies oocyte nutrients. Second, follicle development requires the GC wall to remain intact, and FSH-facilitated glutamine synthesis can stabilize the GC wall by preventing GC apoptosis, which initiates ovulation starting from FF-exposed GCs. Third, glutamine is an ideal ovulation signal because it can be removed at pre-ovulation, by both the decline of FSH levels and by the consumption of matured human oocytes, which are as large as 100–120 µm at maturation (Bae and Foote, 1975) and are effective glutamine scavenger. Thus, glutamine connects hormone, metabolic, and oocyte/follicle maturation signals to regulate oocyte maturation and ovulation (Fig. 2K).

Previous observations support the role of FF glutamine in ovulation. LH promotes glutamine metabolism in oocytes (Zuelke and Brackett, 1993) and may promote ovulation by facilitating FF glutamine depletion. Moreover, many PCOS-related traits are associated with altered glutamine metabolism. For example, androgen signaling promotes glutamine uptake (White et al., 2017), dysregulated insulin signaling (Vuguin et al., 1999) and IGF-I stimulates glutamine uptake in cells cultured in glutamine-starved environments (Wasa et al., 2001). These glutamine uptake-enhancing factors may provide additional support for the hypothesis that high glutamine levels promote PCOS, which was confirmed in our mouse model, and by that although glutamine ameliorates inflammation in DHAE-induced PCOS rats, it failed to ameliorate PCOS histology in them (Wu et al., 2020). Our findings also suggest that some PCOS traits may be more consequential than causal. For example, hyperandrogenemia in PCOS may be induced by the retention of theca cells, which synthesize androgen (Lischinsky and Armstrong, 1983), on unruptured follicles; thus, decreased luteum, whose formation requires ovulation (Devoto et al., 2009), is expected to be a consequence of anovulation.

We reveal that the extrinsic apoptotic pathway, i.e. ASK1-JNK apoptotic pathway, mediates ovulation, and inhibition in ASK1-JNK pathway, such as by elevated FF glutamine, promotes PCOS. This adds to the intrinsic apoptotic pathway functions in follicular atresia for dominant follicle selection (Tilly, 2001) for apoptosis to program oocyte/follicle development/maturation. That ovulation starts from apoptosis of FF-exposed interior GC follicle wall and that GC apoptosis is specifically regulated by the ASK1-JNK apoptotic pathway provides translation values for treating anovulatory diseases such as PCOS. We verified this possibility in mice by showing that metformin, an apoptosis inducer via ASK1-JNK pathway (Feng et al., 2014; Ma et al., 2019), induces ovulation (Barbieri, 2003; Nestler, 2008) and alleviates PCOS as documented (Wang et al., 2020), and these effects can be recaptured by ASK1-JNK apoptotic pathway inducer AT-101 in both glutamine- and EsR1-KO-induced PCOS mouse models. These highlight that GC-specific, potent, and less toxic ASK1-JNK apoptotic inducers can be explored to be novel anti-PCOS drugs.

## Materials and methods

### Antibodies and reagents

The antibodies used in this research were purchased from the following sources: ASK1 (Cell Signaling Technology, Cat. No. 3762), p-ASK1 (Cell Signaling Technology, Cat. No. 3765), JNK (Cell Signaling Technology, Cat. No. 9252), p-JNK (Cell Signaling Technology, Cat. No. 4668), P38 (Cell Signaling Technology, Cat. No. 9212), p-P38 (Cell Signaling Technology, Cat. No. 9211), Caspase3 (Cell Signaling Technology, Cat. No. 9662), Cleaved-Caspase3 (Cell Signaling Technology, Cat. No. 9661), Bcl-XL (Cell Signaling Technology, Cat. No. 2764), LC3B (Cell Signaling Technology, Cat. No 3868), Bcl-2 (ABclonal, Cat. No. A0208), p-RIPK1(S166) (ABclonal, Cat. No. AP1115), RIPK1 (ABclonal, Cat. No. A19580), pMLKL(S358) (ABclonal, Cat. No. AP1244), MLKL (ABclonal, Cat. No. A13451), RIPK3 (ABclonal, Cat. No. A5431), QARS (Proteintech, Cat. No. 12645-1-AP), HA (Abmart, Cat. No.M20003), ACTIN (GeneScript, Cat. No. A00702), GPX4 (Abcam, Cat. No. ab125066), GS (Proteintech, Cat. No. 11037-2-ap), and α-SMA (Cell Signaling Technology, Cat. No.19245).

The reagents employed in this study were purchased from the following companies: Glutamine (Sigma-Aldrich, Cat. No. E6627), FSH (Sigma, Cat. No. F4021 and Solarbio, Cat. No. F8470), LH (Sigma, Cat. No. L6420 and Solarbio, Cat. No. L8040), β-Estradiol (Sigma, Cat. No. E8875), KR33493 (MCE, Cat. No. HY-100755), R7050 (MCE, Cat. No. HY-110203), AT-101 (Selleck, Cat. No. S2812), Gefitinib (MCE, Cat. No. HY-50895), Erlotinib (MCE, Cat. No. HY-50896), hFSH-β-(33-53) (MCE, Cat. No. HY-P3343A), and ^13^C_6_-Glucose (Sigma, Cat. No. 389374).

### Sample collection

This study was approved by the ethics committee of the Obstetrics & Gynecology Hospital of Fudan University and the Shanghai Ji’ai Genetics & IVF Institute (JIAI E2021-020).

#### Human samples

All patients provided written informed consent prior to participation. A total of 72 female patients aged between 23 and 41 (Materials and methods Table S1) were enrolled at the Shanghai Ji’ai Genetics & IVF Institute. Thirty-five of these women were clinically diagnosed as having PCOS and were assigned to the PCOS group, whereas 37 age-matched patients who exhibited normal ovarian function but required IVF treatment for other reasons, such as male partner infertility and uterine or cervical abnormalities, were assigned to the control (CON) group. The two groups were balanced with respect to their body mass index (CON vs. PCOS: 22.07 ± 0.47 vs. 22.73 ± 0.51). Ovarian stimulation and egg retrieval were conducted in accordance with standard IVF guidelines. First, the eggs were picked for IVF purposes, then, FF samples from the same patient were combined and centrifuged to separate the FF and floating granulosa cells before they were subjected to liquid chromatography with tandem mass spectrometry (LC–MS/MS) analysis and cell death assays, respectively.

#### Porcine FF collection

Porcine ovaries obtained from prepubertal gilts at a local slaughterhouse were transported to the laboratory in phosphate-buffered saline at 37°C within 1 h. The follicular fluid was aspirated from superficial follicles with an insulin syringe. FF samples from the same pig were categorized as two samples according to the diameter of the origin follicle (3–8 mm in diameter as antral follicles, larger than 8 mm as pre-ovulatory follicles). All samples were centrifuged to separate the FF and floating granulosa cells before they were subjected to LC–MS/MS analysis and Western blot analysis, respectively.

### Unbiased metabolomic analysis

First, 50 μL of FF or serum was mixed with 200 μL of cold methanol, then centrifuged at 12,000 ×*g* for 30 min at 4°C. The supernatant was filtered with 0.22 µm of polytetrafluoroethylene membrane and transferred to a fresh glass vial for LC–MS/MS analysis. LC–MS/MS analyses were performed using an ultra-high-performance liquid chromatography system (Vanquish, Thermo Fisher Scientific) with an ACQUNITY UPLC BEH Amide column (1.7 µm, 2.1 mm × 100 mm) coupled to a Q Extractive HFX mass spectrometer (Qrbitrap MS, Thermo Fisher Scientific). The mobile phase consisted of (A) 0.3% formic acid and 2 mmol/L ammonium formate in water, and (B) 95% acetonitrile plus 5% water (containing 0.3% formic acid and 2 mmol/L ammonium formate). The analysis was performed by gradient elution at a speed of 0.4 mL/min. The injection volume was 1 μL and the column temperature was 40°C. The data were analyzed using Agilent MassHunter Profinder according to the metabolomic molecular weight, retention time, *m*/*z*, and peak area.

### Murine follicle culture and *in vitro* ovulation

*In vitro* murine follicle culture and ovulation was carried out as described (Skory et al., 2015; Xu et al., 2015). Briefly, ovaries from 14- to 16-day-old mice were cut into four pieces and incubated in α-MEM medium containing 0.1% collagenase I and 0.02% DNase I for 30 min. Enzymatic digestion was quenched with L15 medium containing 1% FBS, and secondary follicles were collected and encapsulated in 0.5% (*w*/*v*) alginate. Follicles were grown in α-MEM medium supplemented with 10 mIU recombinant FSH, 3 mg/mL BSA, 1 mg/mL fetuin, 5 µg/mL insulin, 5 µg/mL transferrin, and 5 ng/mL disodium selenite for 6 days with half of the medium was exchanged every 2 days. Then, alginate was removed with 10 U/mL alginate lyse, and follicles were rinsed with L15 medium containing 1% FBS. The follicular rupture was induced by incubating follicles in glutamine-free α-MEM medium supplemented with 1.5 IU/mL hCG, 5 ng/mL EGF, 3 mg/mL BSA, 5 µg/mL insulin, 5 µg/mL transferrin and 5 ng/mL disodium selenite for 16 h. For glutamine inhibition, glutamine (0 mmol/L, 2 mmol/L, 4 mmol/L or 8 mmol/L) was added, and for FSH inhibition, 1 mmol/L glutamine and FSH (0 mIU, 10 mIU, 20 mIU or 30 mIU) were added.

### Cell apoptotic analysis

Granulosa cells from human FF and cell cultures were treated with a FITC Annexin V Apoptosis Detection Kit I (BD Biosciences, Cat. No. 556547) according to the manufacturer’s instructions. Briefly, granulosa cells were washed twice with phosphate-buffered saline (PBS), and re-suspended with 1 × binding buffer at a density of 1 × 10^6^ cells/100 μL. Then, 5 μL of propidium iodide and 5 μL of Annexin V were added to the solution, which was kept in the dark for 15 min at room temperature. An additional 400 μL of 1× binding buffer was added to stop the reaction and the samples were analyzed using a flow cytometer (BD Biosciences).

For the terminal deoxynucleotidyl transferase dUTP nick-end labeling (TUNEL) assay, granulosa cells from human FF were washed twice with d_2_H_2_O and re-suspended in d_2_H_2_O at a density of 1 × 10^6^ cells/mL. Then, 10 μL of cells was added and adhered on a poly-L-lysine coated slide. The cells or mouse ovary sections were then fixed with 4% paraformaldehyde solution for 15 min, followed by permeabilization with 0.2% Triton X-100 for 10 min at room temperature. Apoptotic cells were identified using the DeadEnd^TM^ Fluorometric TUNEL system (Promega, Madison, WI) according to the manufacturer’s instructions. Briefly, slides were incubated with the enzyme terminal deoxynucleotidyl transferase, recombinant at 37°C for 60 min, then incubated with 2 × SSC buffer for 15 min at room temperature to stop the reaction. Then, the nuclei were stained with DAPI and the slides were mounted with ProLong Glass Antifade Mountants (Thermo Fisher Scientific). Finally, mounted slides were subjected to confocal imaging using a Zeiss LSM710 system.

### Effect of glutamine on apoptosis inhibition in granulosa cells

COV434 cells were cultured in RPMI 1640 medium (Gibco) supplemented with 10% fetal bovine serum (Gibco), KGN cells (the steroidogenic human ovarian granulosa-like tumor cell line) were cultured in DMEM/F12 medium (Hyclone) supplemented with 10% fetal bovine serum (Gibco). All cells were cultured in an incubator with 5% CO_2_ at 37°C. siRNA analysis and plasmid transfection were performed with Lipofectamine 3000 reagent (Thermo Fisher Scientific) according to the manufacturer’s instructions. siRNA was used at a concentration of 20 nmol/L, with 1 µg of plasmids for each well in a six-well plate.

Glutamine treatment in the cells was achieved by incubating cells in glutamine-free RPMI 1640 (Gibco) without serum, supplemented with different concentrations of glutamine, for 6 h before harvesting, as indicated in each experiment. For R7050 and KR33493 treatment, cells were incubated in glutamine-free RPMI 1640 without serum for 6 h before adding R7050 or KR33493, with the same concentration of R7050 or KR33493 added again after 1 h. Cells were then cultured for another hour and harvested for Western blot analysis.

### Glutamine assays

For intracellular glutamine measurement, primary GCs, KGN, and COV434 cells were treated with 100 mIU/mL FSH, 100 mIU/mL LH, 1 µmol/L β-estradiol, or 100 mIU/mL FSH plus 1 µmol/L gefitinib, 1µmol/L erlotinib, and 200 ng/mL hFSH-β-(33–53) for 24 h, respectively. Cells were harvested and intracellular glutamine was determined using glutamine/glutamate-Glo assay (Promega, Cat. No. J8021). The data were normalized to the protein concentration, which was measured using BCA assay first, then normalized to the control group again to get relative glutamine levels.

To measure glutamine secreted to the culture media, primary GCs, KGN and COV434 cells were cultured in medium with ^13^C_6_-glucose for three generations, then cultured in regular medium with 100 mIU/mL FSH, 100 mIU/mL LH, 1 µmol/L β-estradiol, or 100 mIU/mL FSH plus 1 µmol/L gefitinib, 1µmol/L erlotinib, and 200 ng/mL hFSH-β-(33-53) for 24 h, respectively. The concentrations of ^13^C-labeled glutamine in the medium were qualified using LC/MS (Thermo Q Exactive HF). The data were normalized to the protein concentrations of the cells, which were measured using BCA assay first, then normalized to control group again to get relative glutamine levels.

### Western blot analysis

Western blot was performed following standard protocols. Briefly, cells were harvested with a loading buffer containing 50 mmol/L Tris–HCl pH 6.8, 10% glycerol (*v*/*v*), 2% SDS (*w*/*v*), 4% β-mercaptoethanol (*v*/*v*), and 0.0012% bromophenol blue (*w*/*v*). For the analysis, each sample was subjected to SDS-PAGE and transferred to nitrocellulose membranes (GE Healthcare Life Science). The membranes were blocked in 5% (*w*/*v*) skim milk in Tris-buffered saline with 0.1% (*v*/*v*) Tween-20 (TBST) for 1 h at room temperature, then probed overnight with primary antibodies in an antibody dilution buffer (QuickBlockTM, Beyotime) at 4°C. After incubation with horseradish peroxidase-conjugated secondary antibodies in TBST (containing 5% skim milk), membranes were developed using ECL-Plus (Thermo Fisher Scientific) and visualized using Typhoon (GE Healthcare Life Science). All the Western blot experiments were biologically repeated at least three times.

### High-glutamine chow mouse PCOS model

All animal procedures were conducted in accordance with the animal care committee at Fudan University. Female C57BL/6J mice were obtained from Shanghai SLAC Laboratory Animal Co., Ltd. (Shanghai, China). Mice were housed in a specific pathogen-free facility at 20–22°C on a 12-h light/dark cycle with ad libitum access to food and water. Mice were randomly divided into three groups. One group was fed with standard chow (CON), and the other two groups were fed with 30% glutamine chow (GLN) or 30% threonine chow (THR), respectively, both of which were manufactured by FBSW Biotechnology Co., Ltd. The timeline for the mice experiments is illustrated in Fig. S4A. Estrogen receptor 1 knockout (EsR1-KO) mice were purchased from Shanghai Model Organisms.

### Serum analysis

The blood samples were collected from the submandibular vein and centrifuged at 3,000 rpm at 4°C or 15 min and stored at −80°C for subsequent serum determination. The levels of testosterone, estradiol, luteinizing hormone, and follicle-stimulating hormone were determined by ELISA assay kits for mice.

### ITT and GTT analysis

Assays were performed on female C57BL/6J mice. For GTT analysis, mice were intraperitoneally injected with glucose (1 g/kg) after 16 h of fasting, and the blood was sampled 0, 15, 30, 60, 90, and 120 min after glucose injection. For ITT analysis, mice were intraperitoneally injected with 0.4–0.5 units/kg of insulin after 6 h of fasting, and the blood was sampled 0, 15, 30, 60, 90, and 120 min after insulin injection.

### Mice treatments

For FSH and hFSH-β-(33-53) treatments, 8 weeks-old female C57BL/6J mice were randomly divided into sub-groups; and received intraperitoneal injection of 1 Unit/mice of FSH, or 1 Unit/mice of FSH plus 100 µg/mice of hFSH-β-(33-53), mice injected with saline were used as controls. After 8 h of injection, mice were sacrificed and the ovaries were harvested for immunostaining.

For drug treatments, 6 weeks-old female C57BL/6J mice were randomly divided into sub-groups; and received oral gavage of AT-101 (10 mg/kg/day), or intraperitoneal injection of KR33493 (2.5 mg/kg/day), GS4997 (2.5 mg/kg/day), and R7050 (2.5 mg/kg/day) continuously for 2–3 weeks. The mice were then sacrificed, and the ovaries and blood samples were harvested for staining or histological/biochemical assays, respectively.

### IHC staining

Mice ovaries were harvested and fixed in 4% paraformaldehyde in PBS for 4 h. For hematoxylin and eosin (H&E) staining, ovaries were dehydrated using an ethanol gradient from 50% to 100% and cleared in xylene, then embedded in paraffin. Paraffin-embedded ovaries were sectioned to a thickness of 4 µm on a microtome and picked on microscope slides. After drying overnight at 37°C, ovary sections were subjected to H&E staining using the standard protocol, and images were captured with a Leica microscope. For immunostaining, paraformaldehyde-fixed ovaries were immersed in 30% sucrose overnight, embedded in an OCT matrix (Leica), and cut into 5 µm-thick sections. Sections were fixed in 4% PFA at room temperature for 30 min and permeabilized with 0.1% Triton X-100 in PBS for 15 min. Sections were blocked with 5% normal goat serum in PBS for 1 h at room temperature, and incubated overnight with first antibody (1:50 dilution in 5% normal goat serum) at 4°C. After washing with PBS, sections were incubated with fluorescent conjugated secondary antibody (1:100) in the dark for 1 h at room temperature. Sections were washed and incubated with α-SMA antibody (1:300 dilution in 5% normal goat serum) in the dark for 2 h at room temperature, then incubated with Alex Flour 488 conjugated goat anti-rabbit (1:500) in the dark for 1 h at room temperature after washing with PBS. Sections were washed again and incubated with DAPI for 5 min to display the nuclei. Sections were then mounted in a fluorescent mounting medium and images were captured using a Zeiss LSM710 confocal laser scanning microscope (Carl Zeiss Microscopy, LLC).

### Statistical analysis

Statistical analysis was performed using GraphPad Prism 8.0 (GraphPad Software, Inc.). The two-tailed Student’s *t*-test was performed for the two-group analysis. Comparisons between groups were performed by the unpaired two-tailed Student’s *t*-test. Pooled results were demonstrated as the means ± SEM Differences were considered statistically significant if the *P* value was less than 0.05. Significance was indicated as follows: *, *P* < 0.05; **, *P* < 0.01; ***, *P* < 0.001.

## Supplementary information

The online version contains supplementary material available at https://doi.org/10.1093/procel/pwad065.

pwad065_suppl_Supplementary_Figures_S1-S6

pwad065_suppl_Supplementary_Tables_S1

pwad065_suppl_Supplementary_Tables_S2

## Data Availability

All data are available upon request. Please contact corresponding authors for data requests.

## Abbreviations

ASK1: apoptosis signal-regulated kinase 1
BCL-2: B-cell lymphoma 2
Bcl-xL: BCL-2 like protein 1
Cl-caspase-3: cleaved caspase-3
E2: Estradiol
dUTP: nick-end labeling
EGFR: epidermal growth factor receptor
EsR1: estrogen receptor 1
Fas: tumor necrosis factor receptor superfamily member 6
FF: follicular fluid
FSH: follicle stimulating hormone
GCs: granulosa cells
GLN: glutamine
GPX4: phospholipid hydroperoxide glutathione peroxidase 4
GTT: glucose tolerance test
GS: glutamine synthetase
ITT: insulin tolerance test
JNK: c-Jun N-terminal kinase
LC3B: microtubule associated protein 1 light chain 3 beta
LH: luteinizing hormone
MLKL: mixed lineage kinase domain-like protein
PCOS: polycystic ovary syndrome
QARS: glutaminyl-tRNA synthetase
RIPK1: receptor interacting serine/threonine kinase 1
T: testosterone
TNFR: TNF-α receptor
THR: threonine
TUNEL: terminal deoxynucleotidyl transferase

## Code availability

No new code was generated in this study. All used software is publicly available.
