# Supplementary material for: Follicle stimulating hormone controls granulosa cell glutamine synthesis to regulate ovulation
Source: Protein Cell. 2024 Jan 3;15(7):512–29. doi: 10.1093/procel/pwad065 (PMC11214834; doi:10.1093/procel/pwad065)
Supplement: pwad065_suppl_Supplementary_Figures_S1-S6 [file pwad065_suppl_supplementary_figures_s1-s6.pdf]

Supplementary Figure Legends

Fig. S1 Analysis of hormones and amino acids in human FF
A, Relative expression of Cl-Caspase-3/T-Caspase-3 (**A**), and p-MLKL/MLKL (**B**) in each sample in Figure 1D was calculated (n=4).
B, Bar graph showed the relative expression of p-RIPK/RIPK (left), GPX4/ACTIN (middle), and LC3B/ACTIN (right), relative to Figure 1D.
C, Amino acid levels in FF of CON and PCOS group were determined by LC-MS/MS analysis, CON: n = 37; PCOS: n = 35.
D-E, Comparison of testosterone (T) **(B)** and estradiol (E2) **(C)** levels in the FF of patients with PCOS and age-matched healthy women (n = 40).

Fig. S2 Glutamine specifically regulates granulosa cell apoptosis
A, Representative flow cytometry images of cell apoptosis after glutamine deprivation for 0 h, 2 h, 4 h, 6 h, 8 h, 12 h and 24 h in COV 434 cells.
B, Flow cytometry analysis of the apoptotic ratio of KGN cells after glutamine deprivation for 0 h, 2 h, 4 h, 6 h, 8 h, 12 h and 24 h.
C-D, Activation of ASK1 and its downstream signaling pathway in KGN cells after glutamine deprivation **(C)**, which can be inhibited by glutamine supplementation at 8 h **(D)**.
E-F, Knockdown **(E)** and overexpression **(F)** of ASK1 in COV434 cells.

Fig. S3 Glutamine signaling regulates granulosa cell apoptosis
A-B, Knockdown **(A)** and overexpression **(B)** of QARS in COV434 cells.
C-E, QARS knockdown removed the ability of glutamine to suppress ASK1 signaling in a dose-dependent **(C)** and time-dependent **(D)** manner, whereas QARS overexpression enhanced the ability of glutamine to suppress ASK1 signaling **(E)** in COV434 cells.
F-G, KR33493, but not R7050, inhibited ASK1 signaling **(G)** and apoptosis **(H)** in KGN cells mediated by glutamine starvation.

Fig. S4 Physiologic behaviors of mice
A, Schematic illustration of the timeline of mice experiments in this study.
B-C, Food consumption **(B)** and weight **(C)** of mice fed with regular chow (CON), high-glutamine chow (GLN), and high-threonine chow (THR).
D-E, Water intake **(D)** and urine excretion **(E)** of CON, GLN and THR mice measured with metabolic cages, n = 6 per group.

Fig. S5 Physiologic characterization of mice under different treatments
A, Hematoxylin and eosin staining of ovaries from representative dihydrotestosterone induced PCOS mice (DHT). Scale bar: 100 μm.
B, Representative estrous cycles of CON, GLN, THR, EsR1-KO and DHT mice.
C, Days to complete one estrous cycle determined for CON, GLN, DHT and THR mice as indicated.
D-E, Reduced serum Acrp30 **(D)** and relatively normal levels of insulin (**E**) in CON and GLN mice.
F, MRI images of CON and GLN mice (upper), and percentage of fat mass (lower left) or lean mass (lower right) in CON and GLN mice (n = 6).
G-H, Hematoxylin and eosin staining **(G)** and oil red O staining **(H)** of the liver in CON and GLN mice. Scale bars: 200 μm.

I-K, Serum levels of E2 **(I)**, LH **(J)**, and FSH **(K)** in CON, GLN and THR mice (n = 15-41).
L-N, Serum levels of E2 **(L)**, FSH **(M)** and LH **(N)** in CON, GLN Chow + and GLN Chow – mice (n = 9-15).

Fig. S6 AT-101 activates granulosa cell apoptosis and ovulation
A-B, Serum levels of T and E2 in CON and mice treated with KR33493, GS4997 and R7050 (n = 6).
C-D, Representative images of TUNEL staining (**C**) and immunofluorescence staining of cleaved Caspase-3 (**D**) of GLN mice ovaries with oral gavage of saline (upper panel, Mock) or AT-101 (lower panel, AT-101). Scale bars: 100 μm.
E-F, Representative images of TUNEL staining (**E**) and immunofluorescence staining of cleaved Caspase-3 (**F**) of EsR1-KO mice ovaries with oral gavage of saline (upper panel, Mock) or AT-101 (lower panel, AT-101). Scale bars: 100 μm.
G-H, Hematoxylin and eosin staining of GLN **(G)** and EsR1-KO **(H)** mice ovaries with oral gavage of saline (upper panel, Mock) or AT-101 (lower panel, AT-101). Scale bars: 100 μm.
I, Serum levels of E2 in CON, GLN, EsR1-KO mice and CON, GLN, EsR1-KO mice treated with AT-101 (n=6).
J-M, Areas under the curve of the GTT **(J)**, ITT **(K)** of CON, GLN, and GLN mice injected with AT-101, and GTT **(L)**, ITT **(M)** of CON, Esr1-KO, and EsR1-KO mice injected with AT-101 were statistical analyzed and presented as bar graph, respectively, relative to Figures 7I-7J and 7L-7M.
N, Images of ovaries of EsR1-KO mice treated with saline (Mock) or AT-101 (AT-101). Decreased hemorrhagic cyst were shown in the ovaries of AT-101 group.
O, Relative serum glutamine levels of CON and EsR1-KO mice measured by LC-MS/MS analysis (n=6 for CON mice; n=7 for EsR1-KO mice).

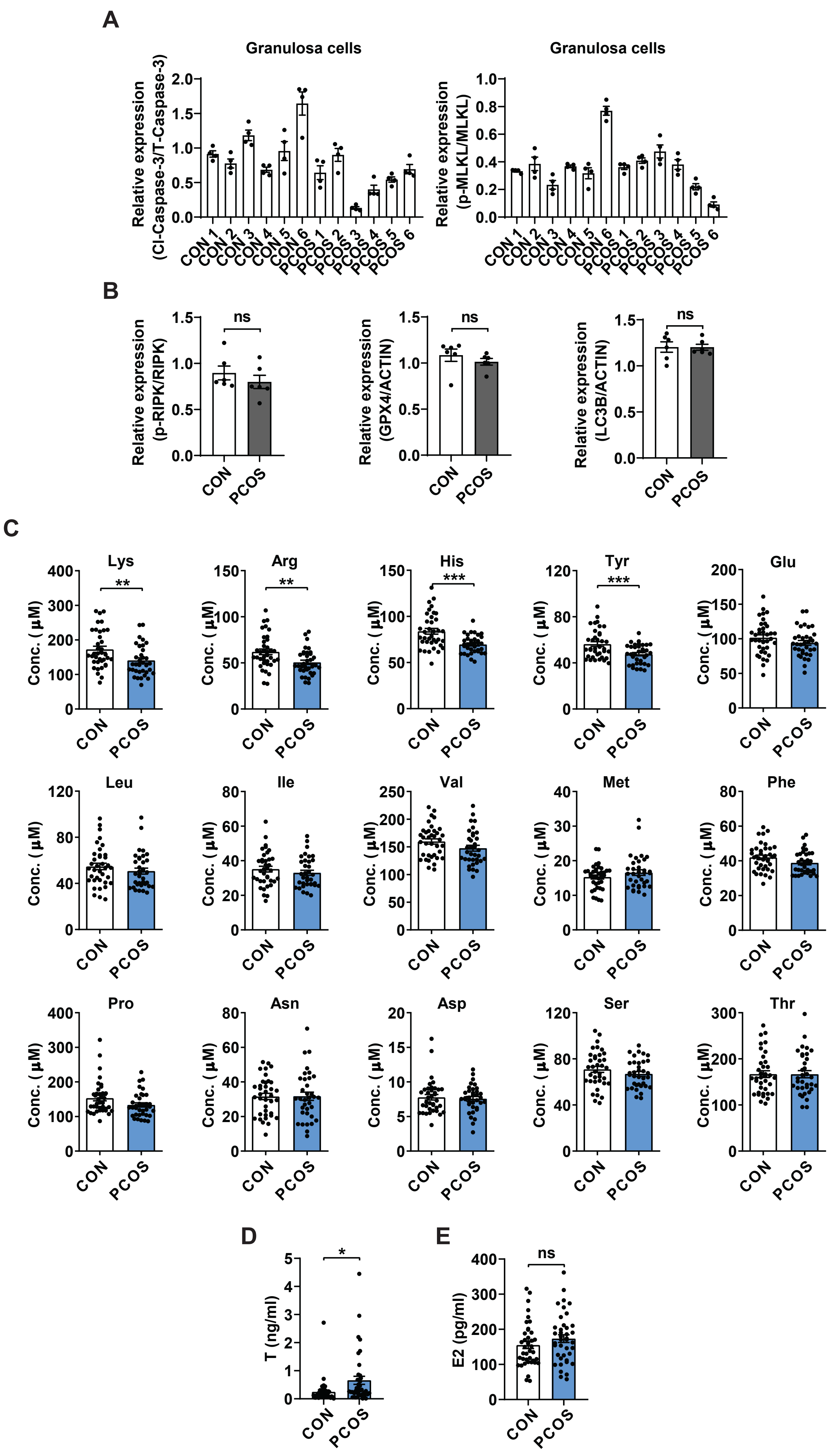
A
Granulosa cells
Relative expression (Cl-Caspase-3/T-Caspase-3)
Relative expression (p-MLKL/MLKL)
CON 1
CON 2
CON 3
CON 4
CON 5
CON 6
PCOS 1
PCOS 2
PCOS 3
PCOS 4
PCOS 5
PCOS 6
B
Relative expression (p-RIPK/RIPK)
Relative expression (GPX4/ACTIN)
Relative expression (LC3B/ACTIN)
ns
CON
PCOS
C
Lys
Arg
His
Tyr
Glu
Leu
Ile
Val
Met
Phe
Pro
Asn
Asp
Ser
Thr
Conc. (μM)
**
***
D
T (ng/ml)
*
E
E2 (pg/ml)
ns

Fig. S1

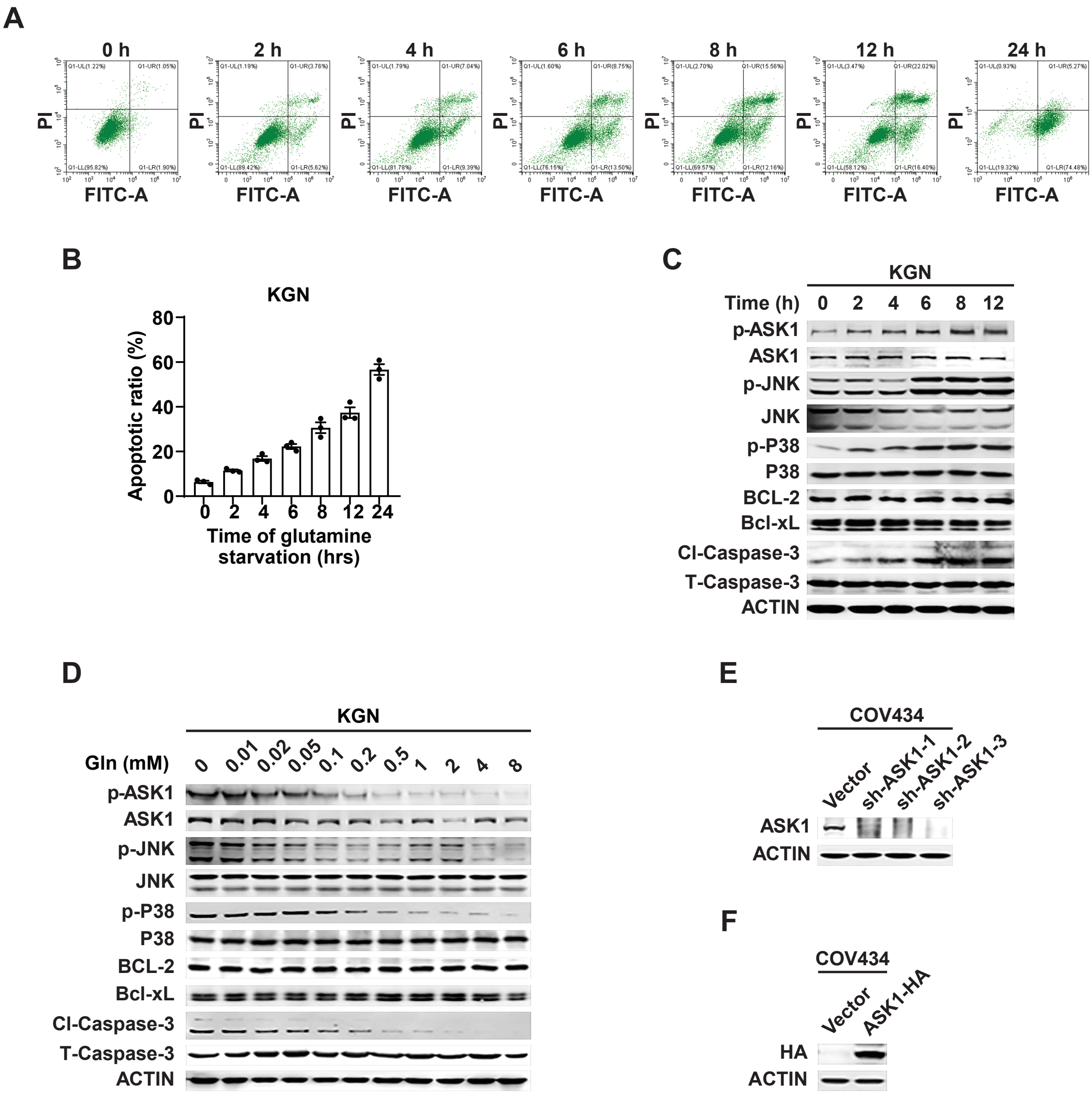
A
0 h
2 h
4 h
6 h
8 h
12 h
24 h
PI
FITC-A
B
KGN
Apoptotic ratio (%)
80
60
40
20
0
0 2 4 6 8 12 24
Time of glutamine starvation (hrs)
C
KGN
Time (h) 0 2 4 6 8 12
p-ASK1
ASK1
p-JNK
JNK
p-P38
P38
BCL-2
Bcl-xL
Cl-Caspase-3
T-Caspase-3
ACTIN
D
KGN
Gln (mM) 0 0.01 0.02 0.05 0.1 0.2 0.5 1 2 4 8
p-ASK1
ASK1
p-JNK
JNK
p-P38
P38
BCL-2
Bcl-xL
Cl-Caspase-3
T-Caspase-3
ACTIN
E
COV434
Vector
sh-ASK1-1
sh-ASK1-2
sh-ASK1-3
ASK1
ACTIN
F
COV434
Vector
ASK1-HA
HA
ACTIN

Fig. S2

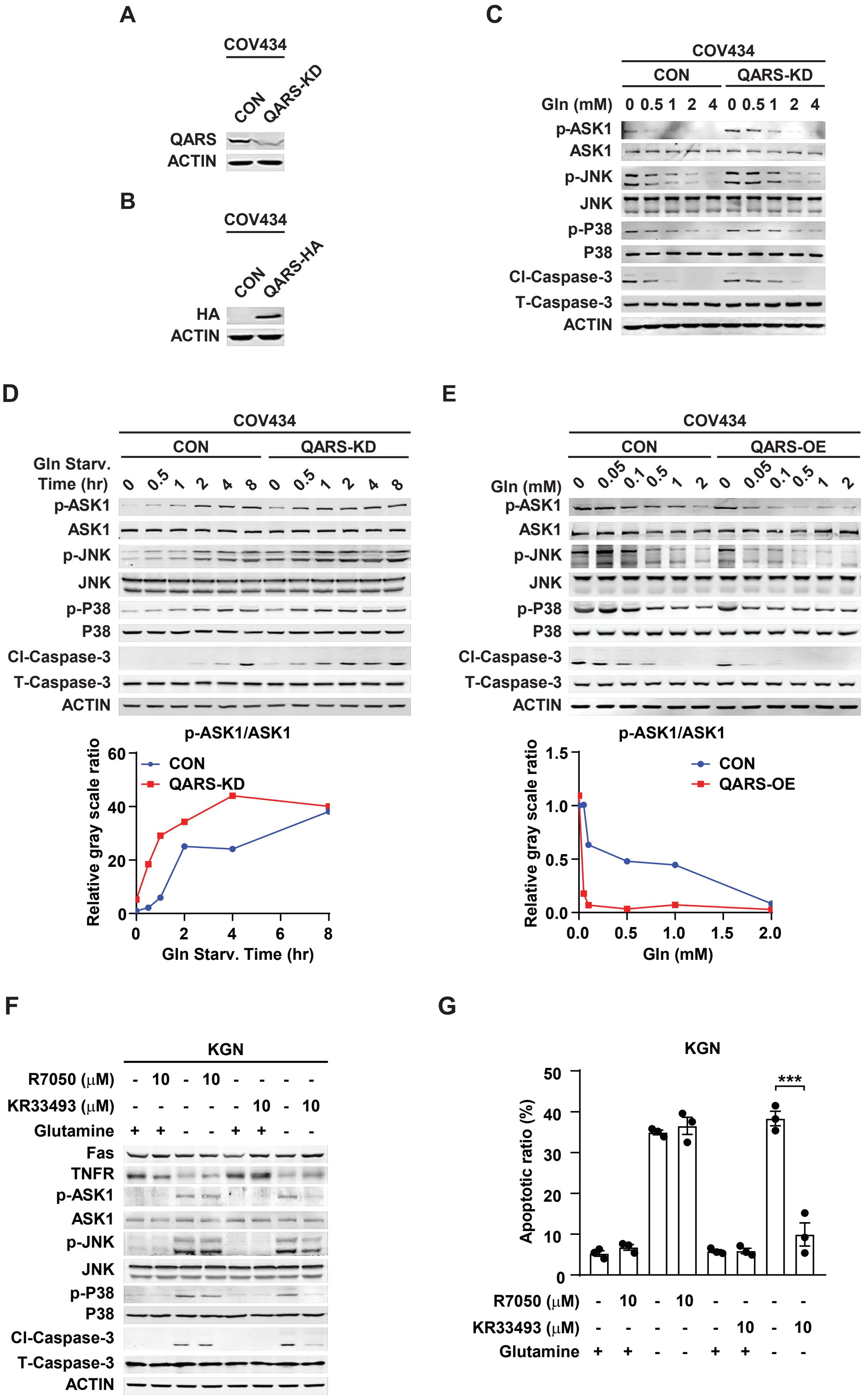

A
COV434
CON
QARS-KD
QARS
ACTIN
B
COV434
CON
QARS-HA
HA
ACTIN
C
COV434
CON
QARS-KD
Gln (mM) 0 0.5 1 2 4 0 0.5 1 2 4
p-ASK1
ASK1
p-JNK
JNK
p-P38
P38
Cl-Caspase-3
T-Caspase-3
ACTIN
D
COV434
CON
QARS-KD
Gln Starv. Time (hr) 0 0.5 1 2 4 8 0 0.5 1 2 4 8
p-ASK1
ASK1
p-JNK
JNK
p-P38
P38
Cl-Caspase-3
T-Caspase-3
ACTIN
p-ASK1/ASK1
Relative gray scale ratio
CON
QARS-KD
Gln Starv. Time (hr)
E
COV434
CON
QARS-OE
Gln (mM) 0 0.05 0.1 0.5 1 2 0 0.05 0.1 0.5 1 2
p-ASK1
ASK1
p-JNK
JNK
p-P38
P38
Cl-Caspase-3
T-Caspase-3
ACTIN
p-ASK1/ASK1
Relative gray scale ratio
CON
QARS-OE
Gln (mM)
F
KGN
R7050 (μM) - 10 - 10 - - - -
KR33493 (μM) - - - - - 10 - 10
Glutamine + + - - + + - -
Fas
TNFR
p-ASK1
ASK1
p-JNK
JNK
p-P38
P38
Cl-Caspase-3
T-Caspase-3
ACTIN
G
KGN
Apoptotic ratio (%)
***
R7050 (μM) - 10 - 10 - - - -
KR33493 (μM) - - - - - 10 - 10
Glutamine + + - - + + - -

Fig. S3

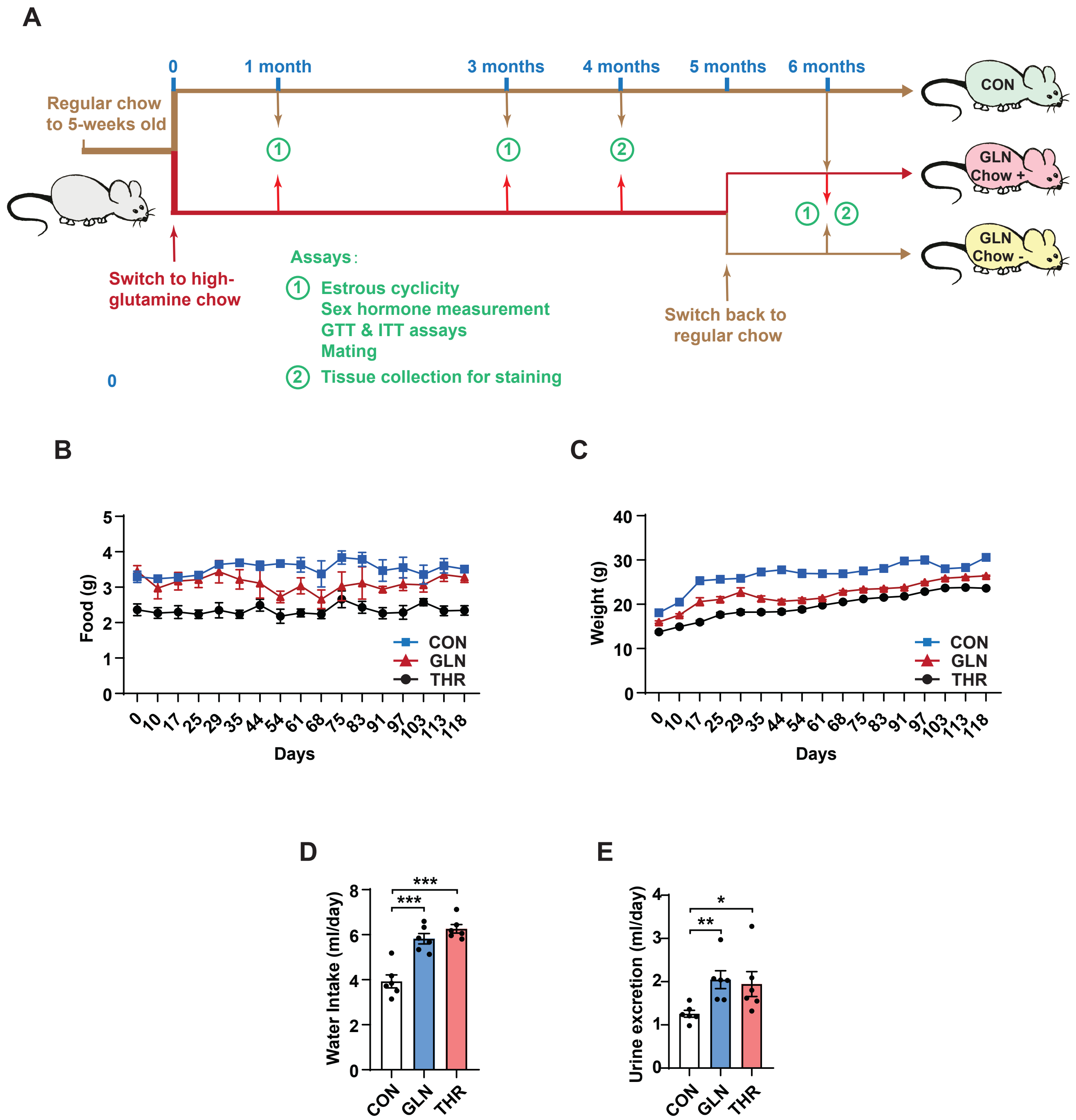
A
0
1 month
3 months
4 months
5 months
6 months
Regular chow
to 5-weeks old
CON
GLN
Chow +
GLN
Chow -
Switch to high-
glutamine chow
Assays:
1 Estrous cyclicity
Sex hormone measurement
GTT & ITT assays
Mating
2 Tissue collection for staining
Switch back to
regular chow
B
Food (g)
Days
CON
GLN
THR
C
Weight (g)
Days
CON
GLN
THR
D
Water Intake (ml/day)
CON
GLN
THR
E
Urine excretion (ml/day)
CON
GLN
THR

Fig. S4

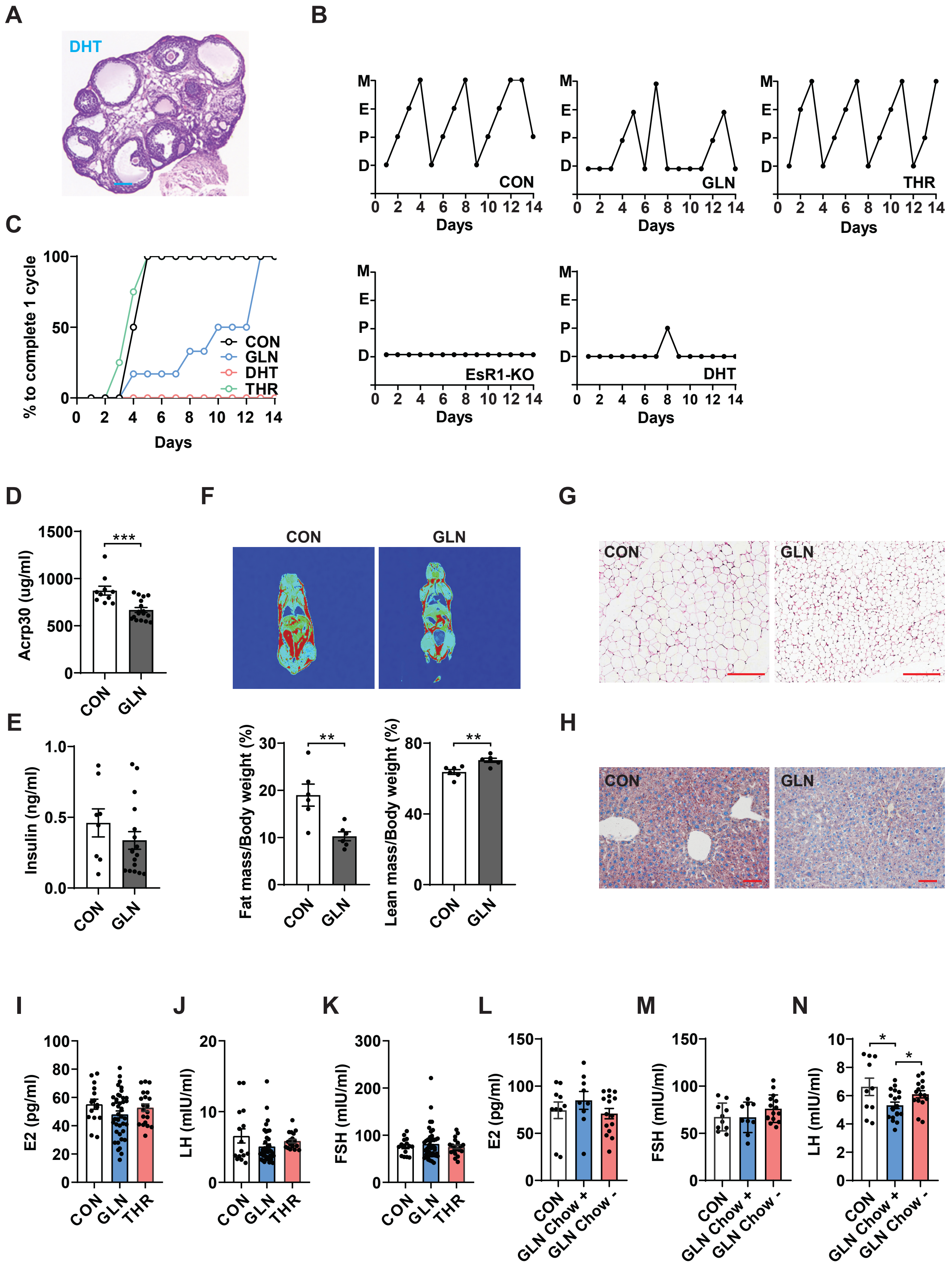
A
DHT
B
CON
GLN
THR
EsR1-KO
DHT
Days
C
% to complete 1 cycle
CON
GLN
DHT
THR
Days
D
Acrp30 (ug/ml)
***
E
Insulin (ng/ml)
F
CON
GLN
Fat mass/Body weight (%)
Lean mass/Body weight (%)
**
G
H
I
E2 (pg/ml)
J
LH (mIU/ml)
K
FSH (mIU/ml)
L
M
N
GLN Chow +
GLN Chow -
THR

Fig. S5

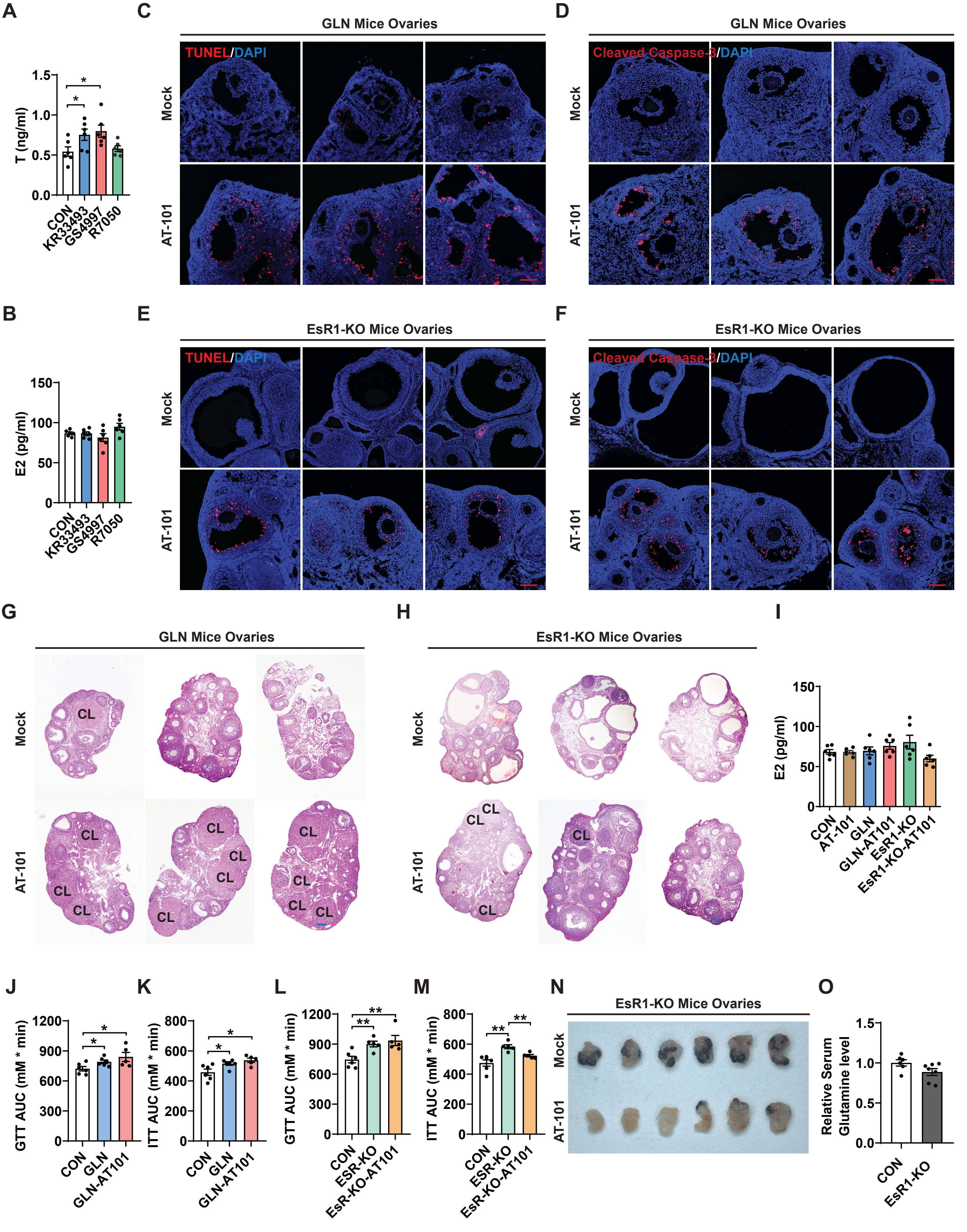
A
T (ng/ml)
CON
KR33493
GS4997
R7050
B
E2 (pg/ml)
C
GLN Mice Ovaries
TUNEL/DAPI
Mock
AT-101
D
GLN Mice Ovaries
Cleaved Caspase-3/DAPI
E
EsR1-KO Mice Ovaries
TUNEL/DAPI
F
EsR1-KO Mice Ovaries
Cleaved Caspase-3/DAPI
G
GLN Mice Ovaries
CL
H
EsR1-KO Mice Ovaries
I
E2 (pg/ml)
CON
AT-101
GLN
GLN-AT101
EsR1-KO
EsR1-KO-AT101
J
GTT AUC (mM * min)
CON
GLN
GLN-AT101
K
ITT AUC (mM * min)
L
GTT AUC (mM * min)
CON
ESR-KO
EsR-KO-AT101
M
ITT AUC (mM * min)
N
EsR1-KO Mice Ovaries
O
Relative Serum Glutamine level
CON
EsR1-KO

Fig. S6
